# Dissemination planning in exercise oncology trials—a systematic review of trial protocols

**DOI:** 10.1007/s00520-025-09532-4

**Published:** 2025-05-15

**Authors:** Emily Smyth, Lydia Politi, Emer Guinan, David Mockler, Linda O’Neill

**Affiliations:** 1 Discipline of Physiotherapy, Trinity College Dublin, Dublin, Ireland; 2 Trinity St James’s Cancer Institute, Dublin, Ireland; 3 School of Biochemistry and Immunology, Trinity College Dublin, The University of Dublin, Dublin, Ireland; 4 John Stearne Library, Trinity Centre for Health Sciences, St. James’s Hospital, Dublin, Ireland; 5 Clinical Research Centre, School of Medicine, University College Dublin, Dublin, Ireland

**Keywords:** Dissemination, Exercise oncology, SPIRIT 2013 checklist

## Abstract

**Purpose:**

The paucity of exercise rehabilitation services for cancer survivors indicates a research-to-practice gap. Dissemination and Implementation research addresses this gap by focusing on the adoption, implementation, and sustainability of evidence-based interventions. Dissemination, the active process of sharing research findings, is critical to the implementation of evidence-based practice. This systematic review examined adherence of exercise oncology trial protocols to the SPIRIT 2013 checklist items pertaining to dissemination planning, items 31a, 31b, and 31c, which address how dissemination is planned, authorship eligibility is considered, and what plans are in place to share data and the protocol.

**Methods:**

A systematic review was conducted following the PRISMA guidelines. EMBASE, MEDLINE, CINAHL, Web of Science—Core Collection, Google Scholar, and the Central Trial Registry via Cochrane were searched (16/05/2024). Title and abstract screening, full-text review, and data extraction were completed in duplicate.

**Results:**

Eighty-six trial protocols were included, thirty-one (36.1%) did not report dissemination plans. Item 31 was reported as follows (*n* = number of trials, frequency (%)); 31a plans to communicate trial results to: participants (*n* = 19, 22.1%), healthcare professionals (*n* = 43, 50%), the public (*n* = 25, 29.2%), and other relevant groups (*n* = 22, 25.6%), 31b: author eligibility (*n* = 3, 3.5%) and plans regarding use of professional writers (*n* = 4, 4.7%), and 31c plans for granting access to participant level dataset (*n* = 28, 32.6%), full protocol (*n* = 1, 1.2%) and statistical code (*n* = 1, 1.2%). Peer-reviewed journal (*n* = 41, 47.67%) and conferences/professional meetings (*n* = 38, 44.2%) were the most frequently reported planned dissemination strategies.

**Conclusion:**

Reporting of the SPIRIT 2013 checklist Item 31 is generally low in exercise oncology trial protocols. Greater consideration of dissemination planning is required to support the implementation of exercise oncology research into practice.

**Registration:** https://doi.org/10.17605/OSF.IO/M8HFP

**Supplementary Information:**

The online version contains supplementary material available at 10.1007/s00520-025-09532-4.

## Background

The incidence of cancer worldwide is rapidly growing. An estimated 20 million new cases were reported in 2022, with a projected increase of 77% by 2050 [1]. Cancer is associated with significant morbidity and mortality, leading to substantial global burden [1, 2]. Multiple treatment options are available for cancer; however, many are associated with significant side effects impacting health-related quality of life (HRQOL) and functional capacity [3]. With a growing number of people living with and beyond cancer worldwide, there is a need for targeted interventions which reduce symptom burden and enhance HRQOL. Exercise has been shown to be safe and feasible across the cancer care continuum [3, 4]. Exercise is associated with a reduced risk of mortality and morbidity and is an effective strategy to relieve many of the side effects of cancer and its treatments [3, 5]. In recent years the volume of research in this area has grown, with clear guidelines for exercise prescription in place for cancer survivors [3].

Despite strong evidence supporting the role and benefits of exercise interventions, exercise services remain limited in oncology, highlighting a research-to-practice gap [5–9]. This research-to-practice gap is neither novel nor unique to exercise oncology trials. Indeed, in the late 1990s, Balas and Boren reported on how it takes approximately 17 years for just 14% of original research to be integrated into patient care [10]. Resultantly, recent decades have seen the emergence of a new science focusing on the acceleration of research findings into clinical care [11]. Dissemination and Implementation (D and I) science aims to enhance the successful adoption, implementation, and maintenance of evidence-based interventions [12]. Dissemination is the active process of sharing research findings [13]. Implementation in contrast focuses on the systematic processes, activities, and resources that are integral to implementing interventions into clinical care and is informed by effective dissemination [12, 14]. Trialists are ethically bound to disseminate their findings. Dissemination demonstrates the responsible use of public funds for research, ensures potential benefits and harms of interventions are shared with stakeholders, and improves access to evidence-based interventions reducing health disparities [14]. In the context of exercise oncology, poor dissemination has been identified as a significant barrier to implementation [15]. Granger et al. reported that lack of evidence and limited knowledge regarding the benefits of exercise for those with cancer amongst the wider multi-disciplinary team are significantly impacting the ability to integrate exercise into cancer care, highlighting the requirement for improved dissemination of exercise oncology trial findings [16]. The effectiveness of dissemination can be improved through systematic, prospective planning, and accordingly dissemination planning should be prioritised at protocol development stage [17, 18].

The SPIRIT (Standard Protocol Items: Recommendations for Interventional Trials) 2013 checklist provides a list of recommended items to address in clinical trial protocols and its associated documents. Adherence to the SPIRIT 2013 checklist enhances trial protocol transparency and robustness for the benefit of all stakeholders [19]. Within the SPIRIT 2013 checklist, dissemination policy is identified as an area for inclusion in clinical trial protocols, with items number 31a, 31b, and 31c focused on how dissemination is planned, how authorship eligibility will be considered, and what plans are in place to share data and the protocol (Table 1).

**Table 1:** SPIRIT 2013 checklist: Item 31 description [19]

SPIRIT 2013 checklist item	Description
31a	Plans for investigators and sponsor to communicate trial results to participants, healthcare professionals, the public, and other relevant groups (e.g. via publication, reporting in results databases, or other data sharing arrangements), including any publication restrictions
31b	Authorship eligibility guidelines and any intended use of professional writers
31c	Plans, if any, for granting public access to the full protocol, participant-level dataset, and statistical code

Therefore, the primary aim of this review was to explore the adherence of exercise oncology trial protocols in reporting of SPIRIT items 31a, 31b, and 31c. A secondary objective was to create an inventory of planned dissemination activities in exercise oncology trials.

## Methods

The systematic review was conducted following the Preferred Reporting Items for Systematic Reviews and Meta-analysis (PRISMA) guidelines [20]. A search strategy was defined using keywords in consultation with the subject librarian (DM). The databases EMBASE, MEDLINE, CINAHL, Web of Science—Core Collection, Google Scholar, and the Central Trial Registry via Cochrane were searched. Search terms included ‘'research protocol'/exp OR'protocol'/de OR'clinical trial protocol'/exp’ OR ‘exercise'/exp OR'physical activity'/exp OR'kinesiotherapy'/exp’ OR ‘'neoplasm'/exp OR'cancer survival'/exp OR'cancer survivor'/exp’ (see Supplementary Material A for full search strategy).

Eligibility criteria were: (i) manuscript must be a protocol paper, (ii) planned study design must be a randomised controlled trial, (iii) planned study population must be adult (aged 18 years and over) cancer survivors, (iv) planned intervention of at least one trial arm must be an exercise intervention which adheres to the FITT prescription principles (frequency, intensity, time and type). For the purpose of this review cancer survivor was defined as ‘*patients with a confirmed diagnosis of cancer from the moment of diagnosis, through the balance of life’* [21]. Exercise was defined as ‘*planned, structured, and repetitive bodily movement which aimed to maintain or improve physical fitness*’ [22]. Exclusion criteria were: (i) protocols published prior to the publication of the SPIRIT 2013 checklist, (ii) protocol not available in English, (iii) full text of protocol unavailable, (iv) protocol registration only, (v) included participants under 18 years of age, and (vi) planned exercise intervention that did not follow FITT principles, was combined with another non-exercise intervention (e.g. behavior change, dietetics), or planned intervention was a physical activity recommendation only.

Title and abstract screening were completed in duplicate by LP and LON using Covidence, a systematic review management system; any disagreements were resolved by a third reviewer EG. Full text review was conducted following the same process. Data was extracted onto a preformatted Excel sheet. Data extraction was completed independently by two reviewers ES and LP, data was compared, and any differences were discussed and resolved with the aid of a third reviewer (LON). Data extracted included planned sample size, cancer type, timepoint of intervention in cancer trajectory, intervention characteristics, and adherence to reporting of item 31a, 31b, and 31c on the SPIRIT guidelines. A repository of planned dissemination strategies in exercise oncology trials was also generated.

## Results

**Fig. 1:**
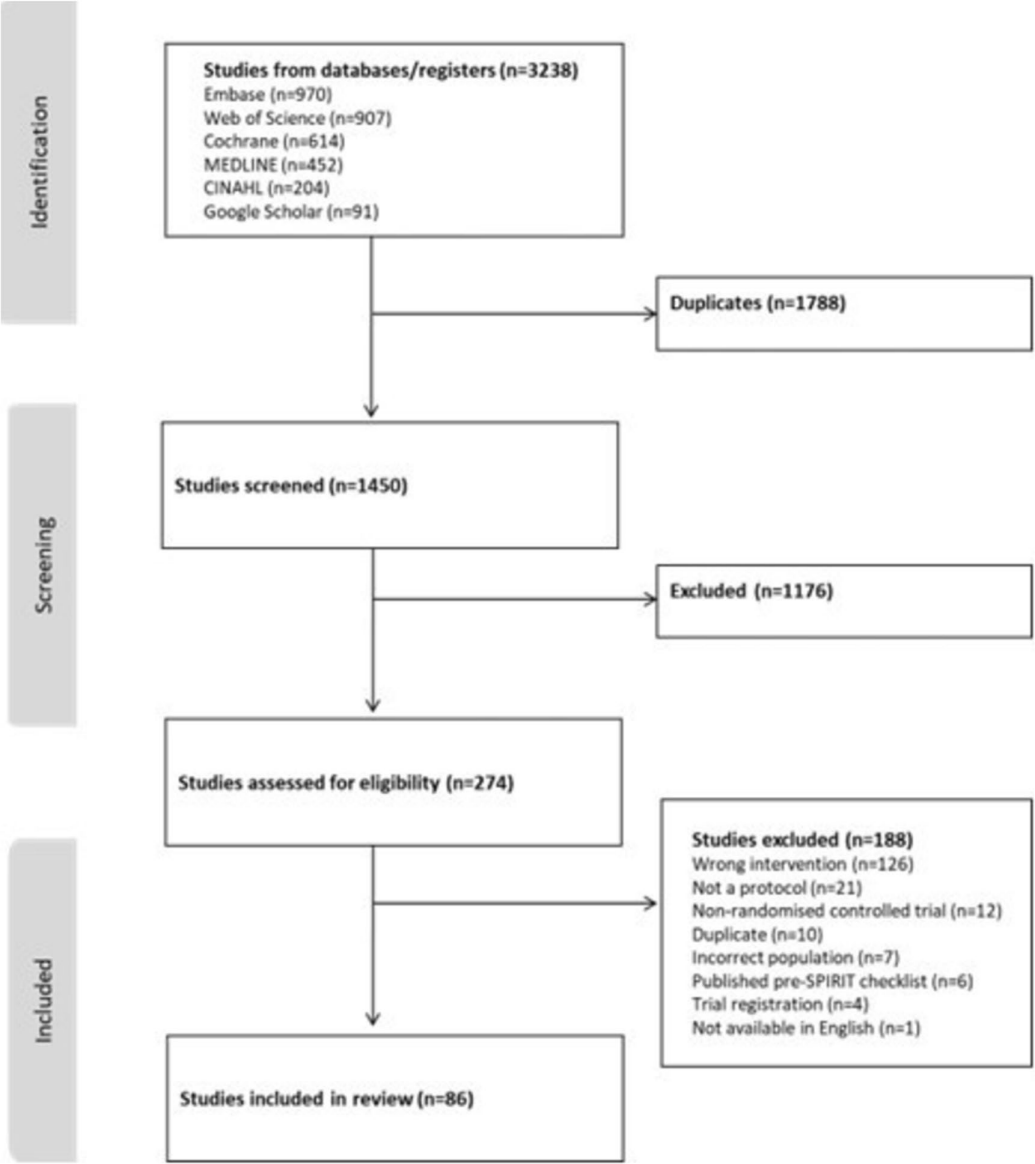
PRISMA diagram

The literature search results are presented in the PRISMA Diagram (Fig. 1). On May 15th 2024, a total of 3238 papers were identified, 1788 were removed due to duplication leaving 1450. Following screening of title and abstracts, 274 full texts were assessed for eligibility. Of the 274 full texts, 188 were excluded due to wrong intervention (*n* = 126), non-protocol publication (*n* = 21), non-randomised controlled trial protocol (*n* = 12), duplicate (*n* = 10), incorrect population (*n* = 7), published prior to publication of the SPIRIT 2013 checklist (*n* = 6), trial registration only (*n* = 4), and not available in English (*n* = 1). In total, *n* = 86 were included in the final review. The reference list and reason for exclusion of reports at full text review stage are included as Supplementary Material B.

The full list of included trial protocols and their demographic details are presented in Supplementary Material C. Trial Protocol papers were published between 2014 and 2024. Planned trials were due to take place in Europe (*n* = 38, 44.2%), North America (*n* = 20, 23.3%), Australia and New Zealand (*n* = 11, 12.8%), United Kingdom (*n* = 8, 9.3%), Asia (*n* = 5, 5.8%), and South America (*n* = 4, 4.65%). Most trial protocols planned to examine patients diagnosed with breast cancer (*n* = 36, 41%), multiple cancer types (*n* = 10, 11.6%), and prostate cancer (*n* = 8, 9.3%). Planned sample size ranged from *n* = 20 to *n* = 1560. Of the 86 protocols, *n* = 8 (9.3%) planned to deliver exercise interventions during the prehabilitation phase, *n* = 32 (36%) active treatment, *n* = 31 (30.04%) post treatment completion, and *n* = 15 (17.44%) across multiple timepoints in cancer care trajectory. The majority of planned interventions were aerobic and resistance training interventions (*n* = 41, 47.7%), aerobic exercise (*n* = 15, 17.4%) and high intensity interval training (*n* = 10, 11.6%).

## Adherence to the SPIRIT 2013 checklist Item 31

Details of each individual trial protocol’s adherence to Item 31 of the SPIRIT 2013 checklist item are available in Supplementary Material D. The number of trials adhering to SPIRIT 2013 Checklist items 31a, 31b, and 31c is presented in Table 2. Overall, *n* = 31 (36.05%) did not report any plans for dissemination. For item 31a, *n* = 19 (22.1%) reported plans to communicate overall trial results to participants in the trial. Additionally, *n* = 14 (16.3) reported plans to communicate individual results to participants. In total, *n* = 43 (50%) reported plans to communicate results to healthcare professionals. Less than a third of trial protocols (*n* = 25 (29%)) reported plans to communicate results to the public. Finally, *n* = 22 (25.6%) reported plans to communicate results to other relevant groups, primarily governing bodies (*n* = 6, 6.9%). For item 31b, *n* = 3 (3.4%) reported the use of author eligibility guidelines and *n* = 4 (4.7%) reported on whether they plan to use professional writers or not. For item 31c, *n* = 28 (32.5%) reported plans for granting access to participant level dataset. Of those, *n* = 4 (4.7%) planned open access to participant level dataset. Finally, *n* = 1 (1.2%) presented plans for access to the full protocol and *n* = 1 (1.2%) for granting access to statistical code.

**Table 2:** Adherence to reporting of SPIRIT 2013 checklist Item 31

SPIRIT 2013 checklist: Item 31	Total *n* = 86
Item 31a	
Plans to communicate trial results to participants	*n* = 19 (22.1%)
Plans to communicate trial results to healthcare professionals	*n* = 43 (50%)
Plans to communicate trial results to the public	*n* = 25 (29.2%)
Plans to communicate trial results to other relevant groups	*n* = 22 (25.6%)
Item 31b	
Author eligibility guidelines	*n* = 3 (3.5%)
Intended use of professional writers	*n* = 4 (4.7%)
Item 31c	
Plans for granting public access to the full protocol	*n* = 1 (1.2%)
Plans for granting access to the participant level dataset	*n* = 28 (32.6%)
Plans for granting access to the statistical code	*n* = 1 (1.2%)

*n* = number of trials, percentage frequency

**Fig. 2:**
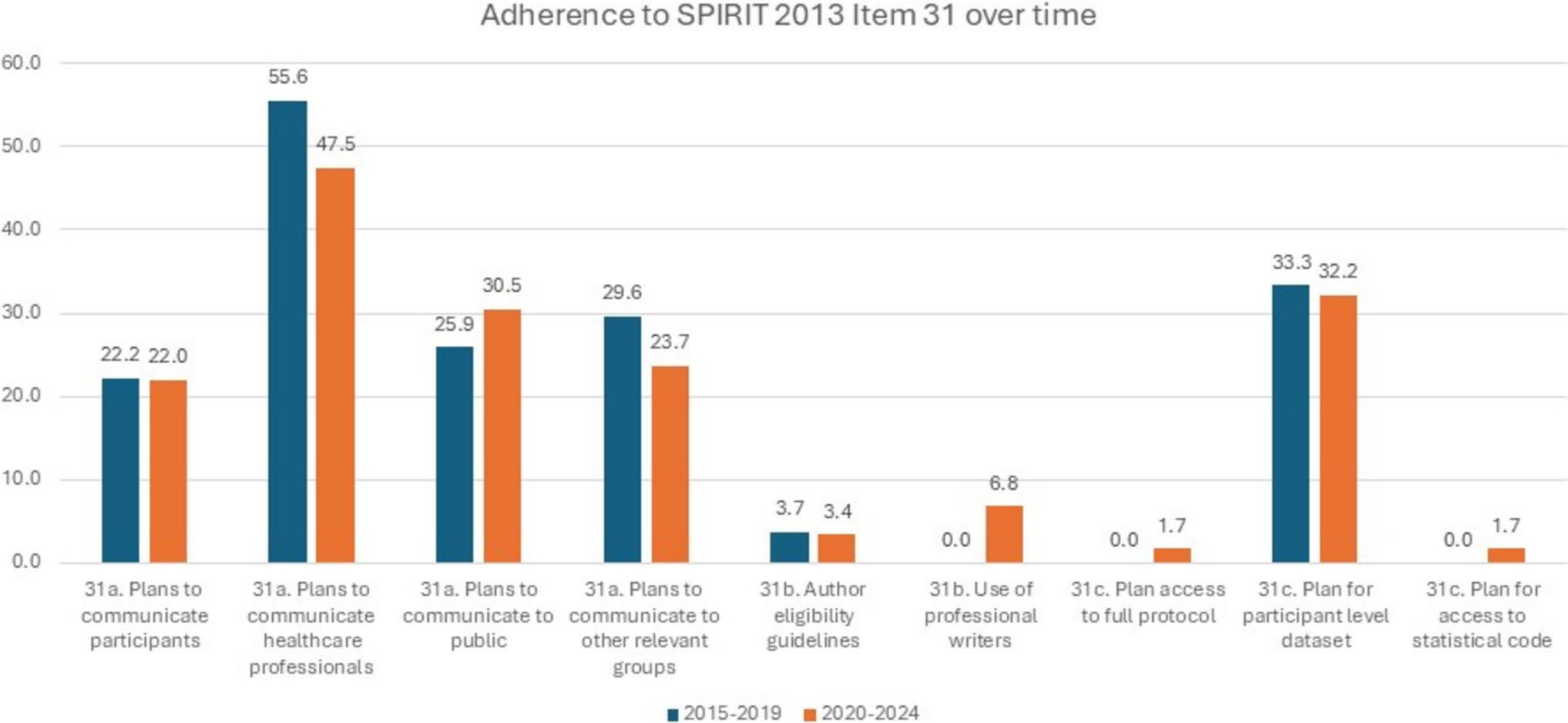
Percentage of studies published between 2015–2019 and 2020–2024 which reported on SPIRIT 2013 checklist Item 31

Table 3 presents adherence to reporting of SPIRIT 2013 checklist Item 31 per geographical region. Clear regional comparisons are difficult due to the variance in number of studies per region. However, studies completed in Europe were most likely to report on all aspects of Item 31, whereas studies completed in South America were the least likely to report on most aspects of Item 31. To examine changes in adherence over time we compared studies included in this review that were published during the period 2015 to 2019 to those published from 2020 to 2024 (Fig. 2). Item 31a (plans to communicate to healthcare professionals (55.6% vs 47.5%), and plans to communicate to other relevant groups (29.6% vs 23.7%)) was more commonly reported during the period 2015–2019, whereas Items 31a (plans to communicate to the public (25.9% vs 30.5%)), 31b (use of professional writers (0% vs 6.8%)), and 31c (plans for access to full protocol and plan for access to statistical code (0% vs 1.7%)) were more commonly reported during the period of 2020 to 2024.

**Table 3:** Adherence to SPIRIT 2013 checklist Item 31, per geographical region

Geographical region (number of studies)	31a. Plans to communicate participants *N* (% adherence)	31a. Plans to communicate healthcare professionals *N* (% adherence)	31a. Plans to communicate to public *N* (% adherence)	31a. Plans to communicate to other relevant groups *N* (% adherence)	31b. Author eligibility guidelines *N* (% adherence)	31b. Use of professional writers *N* (% adherence)	31c. Plan access to full protocol *N* (% adherence)	31c. Plan for participant level dataset *N* (% adherence)	**31c. Plan for access to statistical code** ***N* (% adherence)**
Asia (*n* = 5)	1 (20.0)	4 (80.0)	2 (40.0)	1 (20.0)	0 (0.0)	0 (0.0)	0 (0.0)	1 (0.0)	0 (0.0)
Australia and New Zealand (*n* = 11)	3 (27.3)	6 (54.5)	3 (27.3)	4 (36.4)	0 (0.0)	0 (0.0)	0 (0.0)	3 (27.3)	0 (0.0)
Europe (*n* = 38)	9 (23.7)	19 (50.0)	11 (28.9)	10 (26.3)	3.0 (7.9)	3.0 (7.9)	1.0 (2.6)	17 (44.7)	1 (2.6)
North America (*n* = 20)	2 (10.0)	8 (40.0)	5 (25.0)	3 (15.0)	0 (0.0)	1.0 (5.0)	0.0 (0.0)	3 (15.0)	0 (0.0)
South America (*n* = 4)	0 (0.0)	1 (25.0)	1 (25.0)	1 (25.0)	0 (0.0)	0 (0.0)	0 (0.0)	2 (50.0)	0 (0.0)
UK (*n* = 8)	4 (50.0)	5 (62.5)	3 (37.5)	3 (37.5)	0 (0.0)	0 (0.0)	0(0.0)	2 (25.0)	0 (0.0)

## Repository of planned dissemination strategies

**Fig. 3:**
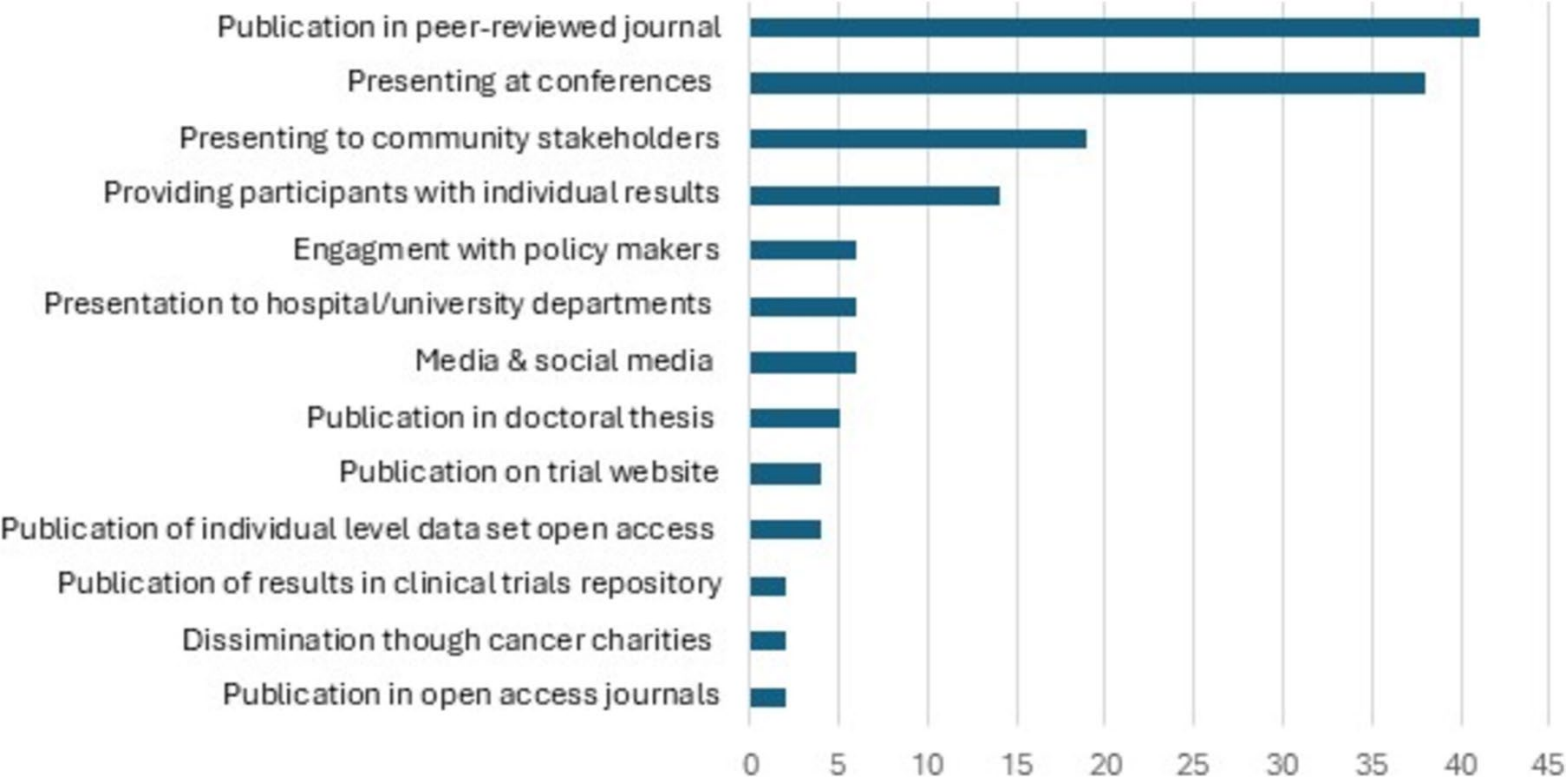
Planned dissemination strategies

Planned dissemination strategies are presented in Fig. 3. Plans to publish in a peer-reviewed journal (*n* = 41, 47.8%) and presentation at conferences, congresses, or professional meetings (*n* = 38, 44.2%) were the most frequently reported planned dissemination strategies. Only *n* = 2 (2.3%) specifically planned to publish results in an open access peer-reviewed journal. The most common approach of communicating results to the public included presenting to community stakeholders (*n* = 19, 22%), use of social media (*n* = 6, 6.9%), and trial website (*n* = 4, 4.7%). Details of dissemination strategies per individual study are provided in Supplemental Material E.

## Discussion

The role of exercise across the cancer care continuum is well established in the research setting [3, 23]. However, the translation of these findings into clinical services is not widespread, limiting the uptake of exercise rehabilitation amongst cancer survivors [24]. Considering the valuable role exercise plays in the management of symptoms, improvement of HRQOL, and reduction of morbidity, enhancing uptake is vital [3]. Improving dissemination of exercise oncology trial findings to aid implementation is an important step towards achieving this goal. The effectiveness of dissemination activities can be improved by planning systematic, stakeholder engaged dissemination from the outset of the research cycle [12]. However, results of this review indicate that dissemination planning is poorly reported across exercise oncology research trial protocols.

Protocol development and publication are key steps in clinical trials and should include plans on dissemination [19, 25, 26]. Despite this, 36.1% of protocols included in this review failed to report plans to disseminate. Failure to adhere to reporting of the SPIRIT 2013 checklist is not uncommon nor is it limited to item 31. The ASPIRE-SCAGE study sought to compare the quality of reporting of RCT protocols from three countries (Canada, Germany and Switzerland) pre to post introduction of the SPIRIT 2013 Checklist [27]. RCT protocols from 2012 (*n* = 257) were compared to 2016 (*n* = 292). The median proportion of reported SPIRIT 2013 checklist items among RCT protocols showed a non-significant increase from 72% (IQR, 63–79%) in 2012 to 77% (IQR, 68–82%) in 2016. Notably, in line with our findings in this review the ASPIRE-SCAGE study identified Item 31c, description of plans for granting access to the full trial protocol, as one of the poorest reported items. Separately the authors of the ASPIRE-SCAGE study also conducted a sub-analysis of the non-regulated trials (of which 11.5% were exercise interventions) included in their review and found that prior to the introduction of the checklist the level of reporting of SPIRIT 2013 checklist items was much lower for non-regulated trials than regulated (59% vs 74%) [28]. However, by 2016 the proportion of reported SPIRIT 2013 checklist items among RCT protocols for non-regulated trials was similar to regulated trials (75% vs 78%). Markedly in relation to dissemination, item 31a was better reported in non-regulated trials in contrast to regulated trials. Findings from the ASPIRE-SCAGE body of work demonstrate that whilst protocol reporting has improved since the introduction of the SPIRIT 2013 checklist, particularly for non-regulated interventions, approximately 25% of SPIRIT checklist items are not being reporting. Similarly in our current review over a third of protocols did not report on item 31 and highlights accordingly that the introduction of the SPIRIT 2013 checklist alone is not a sufficient strategy to enhance protocol reporting.

Suboptimal compliance with the SPIRIT 2013 checklist has also been reported elsewhere. A systematic review by Tan et al. (2020) of 150 randomised controlled trial protocols in humans, across multiple populations and interventions, revealed none of the included protocols reported on all individual checklist items and that an average of 56.7% (95%CI, 54.9 to 58.5%) SPIRIT 2013 checklist items was not adequately reported [29]. In terms of dissemination, Tan et al. reported less than 60% of included protocols reported dissemination plans, whilst guidelines for authorship eligibility and intended use of professional writers were absent in more than 90% of protocols [29], mirroring findings presented in this review. Non-compliance with checklist reporting is not unique to the SPIRIT 2013 checklist. The CONSORT checklist provides a list of minimum items which should be reported when presenting results of completed RCTs [30]; however, adherence to reporting of CONSORT checklist items ranges from 42 to 96% [31]. Whilst evidence supports that the overall reporting quality of RCTs and their protocol papers have improved since the introduction of the CONSORT and SPIRIT 2013 checklists [29, 32], reporting of checklist items in a substantial number of manuscripts remains suboptimal in the field of exercise oncology and beyond. In the context of trial protocols, incomplete and undisclosed reporting of research plans can lead to biases and selective outcome reporting, whereas adherence to the SPIRIT 2013 checklists overwrites issues with completeness and transparency of research plans. However, despite clear guidelines on the SPIRIT 2013 checklist, it is evident that authors are struggling to adhere to all checklist items when preparing manuscripts [19, 33], suggesting a need for greater dissemination and education on the use of the checklist to the research community [29].

This review highlights a strong reliance in exercise oncology trials on traditional dissemination methods such as publication in peer-reviewed journals (*n* = 41, 47.67%) and presentation at conferences/congresses and professional meetings (*n* = 38, 44.19%) [34]. This finding is unsurprising given the value that is placed on these outputs by higher education institutions. Research publications indexed in international databases impact hugely on university global rankings [35], and accordingly these metrics are prioritised in the hiring, tenure, and promotion of academic staff. For example, a review of promotion and tenure documents of 129 North American universities found 90–95% included traditional outputs, e.g. peer-reviewed journals, books or book chapters, conference presentations etc. as part of their assessment criteria [36]. Whilst publication through peer-reviewed journal remains the benchmark for dissemination of trial results to healthcare providers, this passive approach may not support effective and timely communication of results, particularly if journals are not open access [13, 37]. Only two protocols specifically planned to publish in open access journals, potentially limiting access to future trial findings. This finding was surprising given that many funders and higher education institutions now mandate open access publication due to its many benefits including its ability to enhance transparency, accelerate dissemination, expand readership, and increase impact of research findings[38, 39]. However, despite its benefits the uptake of open access publishing has been slow. The biggest drawback of open accessing publishing is the cost responsibilities which are inferred to the authorship and ultimately contribute to a two-tiered publication environment wherein ability to pay and not peer review may be the final decisive factor in whether a manuscript is published, particularly disadvantaging researchers from economically disadvantaged backgrounds [39, 40]. Therefore, consideration must be given to strategies to reduce the financial burden associated with open access journals, support dissemination, and ultimately facilitate integration into clinical practice.

In addition, there is an increasing expectation that datasets and statistical code should be made public to enhance research transparency, facilitate reuse of data, data synthesis, and most importantly integration of knowledge into clinical care [41]. Adherence to the FAIR Guiding Principles of data management and stewardship, i.e. that data should be findable, accessible, interoperable, and reusable, is increasingly a requirement of funding agencies, universities, and other research bodies [41]. Notably, less than a third of trial protocols in our review included details on plans to share the participant level data set, and only one protocol paper reported details on sharing the statistical code. The challenge of adhering to the FAIR Guiding Principles is not a uniquity to exercise oncology trials. Concerns exist amongst the research community regarding the enormous effort required in open data sharing [42], the potential of losing publication opportunities [43], and receiving appropriate credit for research endeavours [44]. Most importantly, in this era wherein data protection is paramount, fears exist regarding the privacy and confidentiality of data that is shared [45]. In order to better support trialists to plan for sharing of data in line with the FAIR Guiding Principles research funders, universities, and other research support organisations need to enhance educational opportunities to alleviate these concerns and provide practical training on data management and stewardship [41, 46].

New avenues for dissemination of results are growing, including online dissemination through social media platforms, websites, podcasts, and use of knowledge brokering [47, 48]. These approaches represent novel and exciting methods of communicating results in healthcare. Social media particularly has huge potential as a rapid mobiliser of research findings in exercise oncology [49]. Weller et al. previously reported how a daily exercise oncology twitter campaign was effective at engaging an international audience and reaching an enormous 499,899 impressions over a 9-month period [50]. Despite this potential, only six of the protocols included in this review reported plans to publish results on social media and four on through an online platform. Considering the volume of research waste, exercise oncology trialists should consider exploration of novel approaches to dissemination in a bid to close the evidence to practice gap.

Communication of results to a diverse group of stakeholders is a vital component of an effective dissemination strategy; however, this is insufficiently addressed in current practices [37, 51]. Only 29.2% of protocols included in this review planned to disseminate to the public, 25.6% to other relevant groups, and 22.1% to study participants. Stakeholders are keen to access information on exercise during cancer, with patients actively seeking information online and through newspaper articles [52, 53]. However, effective communication of exercise oncology results to key stakeholders, including patients and family members, is lacking. This is evident from the limited understanding of the benefits of exercise across the cancer care continuum in cancer patients and their families [15, 54]. In a 2024 mixed methods study, participants had an appreciation of the general benefits of exercise; however, when presented in the context of exercising prior to oncological resection patients and their family members required an introduction and explanation to the programme [54]. Furthermore, Kennedy et al. (2022) found a lack of awareness regarding the safety, value of exercise, and methods for doing so in cancer patients [15]. This presents a challenge for trialists, as communicating results effectively requires addressing the unique needs of each stakeholder group. Therefore, it is evident that additional work is required to identify the most appropriate and beneficial dissemination approaches for each stakeholder group. This is vital to support trialist to engage with key stakeholders including patients, healthcare professionals, policy makers from the start of the research cycle and work together to plan dissemination that is tailored and considered for each stakeholder group [37, 51].

## Limitations of review

This review albeit comprehensive has some limitations. Despite a thorough search strategy to ensure all eligible protocols were included to establish a large dataset of approaches to dissemination in exercise oncology, the strict inclusion criteria excluded protocols not published in English. This may have resulted in the exclusion of valuable protocols providing insight into dissemination plans therefore limiting generalisability of results globally. Additionally, to ensure robust results that accurately reflect the dissemination plans in exercise oncology trials, only randomised controlled trials were included. However, non-randomised controlled trials could offer valuable insights into novel dissemination approaches. Analysis of these non-RCT trials may identify additional and novel approaches which were not captured in this review.

## Conclusion

In conclusion, reporting of item 31 on the SPIRIT 2013 checklist is generally poor in exercise oncology protocols. Additional education and training may be required to support trialists to successfully plan for dissemination and ensure the generation of robust results which can be effectively communicated to all stakeholders to aid the delivery of evidence from exercise oncology trials into clinical practice.

## Supplementary Information

Below is the link to the electronic supplementary material.

Supplementary file A (DOCX 25 KB)

Supplementary file B (DOCX 61 KB)

Supplementary file C (DOCX 95 KB)

Supplementary file D (DOCX 77 KB)

Supplementary file E (DOCX 45 KB)

## Data Availability

No datasets were generated or analysed during the current study.
