# Supplementary material for: Dissemination planning in exercise oncology trials—a systematic review of trial protocols
Source: Support Care Cancer. 2025 May 15;33(6):473. doi: 10.1007/s00520-025-09532-4 (PMC12078369; doi:10.1007/s00520-025-09532-4)
Supplement: Supplementary file 1 — Supplementary file A (DOCX 25 KB) [file 520_2025_9532_MOESM1_ESM.docx]

**Supplementary Material A – Search Results and Strategy**

*Title:* Dissemination Planning in Exercise Oncology Trials - A Systematic Review of Trial Protocols

*Authors*: Emily Smyth ^1,2^, Lydia Politi^3^, Emer Guinan^1,2^, David Mockler^4^, and Linda O’Neill^1,2,5^

*Affiliations*:

^1^ Discipline of Physiotherapy, Trinity College Dublin, Dublin, Ireland

^2^ Trinity St James's Cancer Institute, Dublin, Ireland

^3^ School of Biochemistry and Immunology, Trinity College Dublin, The University of Dublin, Dublin, Ireland

^4^ John Stearne Library, Trinity Centre for Health Sciences, St. James’s Hospital, Dublin, Ireland

^5^Clinical Research Centre, School of Medicine, University College Dublin, Dublin, Ireland

*Corresponding Author*: Dr Linda O’Neill

E-mail: [loneill4@tcd.ie](mailto:loneill4@tcd.ie)

| **Search 16/05/2024** |  |
| --- | --- |
| EMBASE | 970 |
| MEDLINE | 452 |
| CINAHL | 204 |
| Web of Science | 907 |
| Cochrane | 614 |
| Google Scholar | 91 |
| **Total** | **3238** |
| **Duplicate** | **1787** |
| **T and I for Screening** | **1451** |

EMBASE [964]

'research protocol'/exp OR 'protocol'/de OR 'clinical trial protocol'/exp

Protocol*:ti

#1 OR #2

'randomized controlled trial'/exp OR 'randomized controlled trial (topic)'/de

('Randomi?ed controlled trial*' OR RCT OR 'Random allocation' OR 'randomly allocated' OR 'Allocated randomly' OR (allocated NEAR/2 random) OR 'Single blind*' OR 'Double blind*' OR ((treble or triple) NEAR/1 (blind*))):ti,ab,kw

#3 OR #4

'exercise'/exp OR 'physical activity'/exp OR 'kinesiotherapy'/exp

(exercis* OR 'physical activit*' OR physiotherapy):ti,ab,kw

#7 OR #8

'neoplasm'/exp OR 'cancer survival'/exp OR 'cancer survivor'/exp

(neoplasm* OR cancer* OR tumor* OR tumour* OR malignant OR oncology):ti,ab,kw

#10 OR #11

#3 AND #6 AND #9 AND #12

Medline [699]

Clinical Trial Protocols as Topic/

Protocol.ti.

1 OR 2

exp Randomized Controlled Trial/ OR exp Randomized Controlled Trials as Topic/

(Randomi*ed controlled trial* OR RCT OR Random allocation OR randomly allocated OR Allocated randomly OR (allocated adj2 random) OR Single blind* OR Double blind* OR ((treble or triple) adj1 (blind*))).tw.

3 OR 4

exp Exercise/ OR exp Sports/ OR exp Exercise therapy/

(exercis* OR 'physical activit*' OR physiotherapy).tw.

7 OR 8

exp Neoplasms/ OR Cancer Survivors/

(neoplasm* OR cancer* OR tumor* OR tumour* OR malignant OR oncology).tw.

10 OR 11

3 AND 6 AND 9 AND 12

CINAHL [209]

(MH "Research Protocols") OR (MH "Protocols")

TI (Protocol*)

S1 OR S2

(MH "Randomized Controlled Trials+")

TI ("Randomi*ed controlled trial*" OR RCT OR "Random allocation" OR "randomly allocated" OR "Allocated randomly" OR (allocated N2 random) OR "Single blind*" OR "Double blind*" OR ((treble or triple) N1 (blind*))) OR AB ("Randomi*ed controlled trial*" OR RCT OR "Random allocation" OR "randomly allocated" OR "Allocated randomly" OR (allocated N2 random) OR "Single blind*" OR "Double blind*" OR ((treble or triple) N1 (blind*)))

S3 OR S4

MH "Exercise+" OR MH "Physical Activity" OR MH "Sports" OR MH "Therapeutic Exercise+"

TI (exercis* OR "physical activit*" OR physiotherapy) OR AB (exercis* OR "physical activit*" OR physiotherapy)

S7 OR S8

(MH "Cancer Patients") OR (MH "Cancer Survivors") OR (MH "Neoplasms+") OR (MH "Rehabilitation, Cancer")

TI (neoplasm* OR cancer* OR tumor* OR tumour* OR malignant OR oncology) OR AB (neoplasm* OR cancer* OR tumor* OR tumour* OR malignant OR oncology)

S10 OR S11

S3 AND S6 AND S9 AND S12

Web of Science Core Collections [907]

((TI=(protocol*)) AND ALL=((exercis* OR "physical activit*" OR physiotherapy))) AND ALL=((neoplasm* OR cancer* OR tumor* OR tumour* OR malignant OR oncology))

Central Trial Registry searched via Cochrane [614]

[mh "Clinical Trial Protocols as Topic"]

Protocol*:ti

#1 OR #2

[mh "Exercise"] OR [mh "Sports"] OR [mh "Exercise therapy"]

(exercis* OR (physical NEAR/2 activit*) OR physiotherapy):ti,ab,kw

#4 OR #5

[mh "Neoplasms"] OR [mh "Cancer Survivors"]

(neoplasm* OR cancer* OR tumor* OR tumour* OR malignant OR oncology):ti,ab,kw

#7 OR #8

#3 AND #6 AND #9

Google Scholar

protocol exercise|exercising|execised|"physical activity"|physiotherapy neoplasm|cancer|tumor|tumour|malignant|oncology
