# Supplementary material for: Dissemination planning in exercise oncology trials—a systematic review of trial protocols
Source: Support Care Cancer. 2025 May 15;33(6):473. doi: 10.1007/s00520-025-09532-4 (PMC12078369; doi:10.1007/s00520-025-09532-4)
Supplement: Supplementary file 2 — Supplementary file B (DOCX 61 KB) [file 520_2025_9532_MOESM2_ESM.docx]

**Supplementary Material B – List of Excluded Reports and Reasons for Exclusion**

*Title:* Dissemination Planning in Exercise Oncology Trials - A Systematic Review of Trial Protocols

*Authors*: Emily Smyth ^1,2^, Lydia Politi^3^, Emer Guinan^1,2^, David Mockler^4^, and Linda O’Neill^1,2,5^

*Affiliations*:

^1^ Discipline of Physiotherapy, Trinity College Dublin, Dublin, Ireland

^2^ Trinity St James's Cancer Institute, Dublin, Ireland

^3^ School of Biochemistry and Immunology, Trinity College Dublin, The University of Dublin, Dublin, Ireland

^4^ John Stearne Library, Trinity Centre for Health Sciences, St. James’s Hospital, Dublin, Ireland

^5^Clinical Research Centre, School of Medicine, University College Dublin, Dublin, Ireland

*Corresponding Author*: Dr Linda O’Neill

E-mail: [loneill4@tcd.ie](mailto:loneill4@tcd.ie)

**Supplementary Material B – List of Excluded Reports and Reasons for Exclusion**

|  | **Reference** | **Reason for exclusion** |
| --- | --- | --- |
| 1 | Adlard, K.N., et al., Peer support for the maintenance of physical activity and health in cancer survivors: The PEER trial - A study protocol of a randomised controlled trial. BMC cancer, 2019. 19(1). | Wrong Intervention |
| 2 | Akdemir, E., et al., EffectiveNess of a multimodal preHAbilitation program in patieNts with bladder canCEr undergoing radical cystectomy: protocol of the ENHANCE multicentre randomised controlled trial. BMJ open, 2023. 13(3). | Wrong Intervention |
| 3 | Al Onazi, M.M., et al., Decongestive progressive resistance exercise with an adjustable compression wrap for breast cancer-related lymphoedema (DREAM): protocol for a randomised controlled trial. BMJ open, 2022. 12(4). | Wrong Intervention |
| 4 | Alibhai, S.M.H., et al., Protocol for a phase III RCT and economic analysis of two exercise delivery methods in men with PC on ADT. BMC cancer, 2018. 18(1). | Wrong Intervention |
| 5 | Allen, S., et al., A randomised controlled trial to assess whether prehabilitation improves fitness in patients undergoing neoadjuvant treatment prior to oesophagogastric cancer surgery: Study protocol. BMJ open, 2018. 8(12). | Wrong Intervention |
| 6 | Andersen, H.H., et al., Exercise in older women with breast cancer during systemic therapy: Study protocol of a randomised controlled trial (BREACE). BMJ open, 2020. 10(10). | Wrong Intervention |
| 7 | Andersson, M., et al., CANOPTIPHYS study protocol: Optimising PHYSical function before CANcer surgery: effects of pre-operative optimisation on complications and physical function after gastrointestinal cancer surgery in older people at risk—a multicentre, randomised, parallel-group study. Trials, 2023. 24(1). | Wrong Intervention |
| 8 | Arietaleanizbeaskoa, M.S., et al., Implementing Exercise in Standard Cancer Care (Bizi Orain Hybrid Exercise Program): Protocol for a Randomized Controlled Trial. JMIR Research Protocols, 2021. 10(8): p. e24835. | Wrong Intervention |
| 9 | Baguley, B.J., et al., Nutrition therapy with high intensity interval training to improve prostate cancer-related fatigue in men on androgen deprivation therapy: A study protocol. BMC cancer, 2017. 17(1). | Wrong Intervention |
| 10 | Barberan-Garcia, A., et al., Cost-effectiveness of a technology-supported multimodal prehabilitation program in moderate-to-high risk patients undergoing lung cancer resection: randomized controlled trial protocol. BMC health services research, 2020. 20(1): p. 207. | Wrong Intervention |
| 11 | Berkel, A.E.M., et al., The effects of prehabilitation versus usual care to reduce postoperative complications in high-risk patients with colorectal cancer or dysplasia scheduled for elective colorectal resection: Study protocol of a randomized controlled trial. BMC Gastroenterology, 2018. 18(1). | Wrong Patient Population |
| 12 | Bernard, S., et al., Control4Life: A randomized controlled trial protocol examining the feasibility and efficacy of a combined pelvic health rehabilitation and exercise fitness program for individuals undergoing prostatectomy. Contemporary clinical trials, 2024. 139. | Wrong Intervention |
| 13 | Bjerre, E., et al., Effectiveness of community-based football compared to usual care in men with prostate cancer: Protocol for a randomised, controlled, parallel group, multicenter superiority trial (The FC Prostate Community Trial). BMC cancer, 2016. 16(1). | Wrong Intervention |
| 14 | Blarigan, E.L.V., et al., Protocol for a 4-arm randomized controlled trial testing remotely delivered exercise-only, diet-only, and exercise plus diet interventions among men with prostate cancer treated with radical prostatectomy (Prostate 8-II). Contemporary clinical trials, 2023. 125. | Duplicate |
| 15 | Bloomquist, K., et al., A randomized cross-over trial to detect differences in arm volume after low- and heavy-load resistance exercise among patients receiving adjuvant chemotherapy for breast cancer at risk for arm lymphedema: Study protocol. BMC cancer, 2016. 16(1). | Not Randomised Controlled Trial |
| 16 | Boeding, J.R.E., et al., Preoptimisation in patients with acute obstructive colon cancer (PREOCC) – a prospective registration study protocol. BMC Gastroenterology, 2023. 23(1). | Not Randomised Controlled Trial |
| 17 | Boing, L., et al., Pilates and dance to patients with breast cancer undergoing treatment: Study protocol for a randomized clinical trial - MoveMama study. Trials, 2020. 21(1). | Wrong Intervention |
| 18 | Brown, M., et al., A multicomponent exercise trial for advanced prostate cancer: A study protocol. Journal of Physical Activity & Health, 2018. 15(10): p. S221-S222. | Not Randomised Controlled Trial |
| 19 | Bruce, J., et al., Randomised controlled trial of exercise to prevent shoulder problems in women undergoing breast cancer treatment: Study protocol for the prevention of shoulder problems trial (UK PROSPER). BMJ open, 2018. 8(3). | Wrong Intervention |
| 20 | Brunet, J., et al., Exploring the Effects of Yoga Therapy on Heart Rate Variability and Patient-Reported Outcomes After Cancer Treatment: A Study Protocol. Integrative Cancer Therapies, 2022. 21. | Not Randomised Controlled Trial |
| 21 | Bucciarelli, V., et al., The differential effects of a short term aerobic or resistance physical exercise protocol in the improvement of endothelial function and cardiovascular efficiency in women after breast-cancer surgery. European Heart Journal Cardiovascular Imaging, 2017. 18: p. iii24. | Not Protocol Paper |
| 22 | Burnett, C., et al., The Prehabilitation Radiotherapy Exercise, smoking Habit cessation and Balanced diet Study (PREHABS) protocol to explore the feasibility of embedding behavioural modifications into the clinical pathway for patients undergoing radical radiotherapy for lung cancer. BMJ open, 2024. 14(1). | Not Randomised Control Trial |
| 23 | Camargo, R.G., et al., Development of an exercise training protocol for cachectic patients. Supportive Care in Cancer, 2011. 19(2): p. S284-S285. | Not Protocol Paper |
| 24 | Casanovas-Álvarez, A., et al., Prehabilitation in Patients With Breast Cancer Receiving Neoadjuvant Therapy to Minimize Musculoskeletal Postoperative Complications and Enhance Recovery (PREOPtimize): A Protocol for a Randomized Controlled Trial. Physical Therapy, 2023. 103(9). | Wrong Intervention |
| 25 | Cho, D., et al., Study protocol: A lifestyle intervention for African American and Hispanic prostate cancer survivors on active surveillance and their partners. Pilot and Feasibility Studies, 2020. 6(1). | Wrong Intervention |
| 26 | Coletta, A.M., et al., Creatine supplementation and resistance training to preserve muscle mass and attenuate cancer progression (CREATINE-52): a protocol for a double-blind randomized controlled trial. BMC cancer, 2024. 24(1). | Wrong Intervention |
| 27 | Dandekar, S., M. Caru, and K.H. Schmitz, Behavioral physical activity intervention coupled with standard post-cancer directed treatment care to mitigate chronic pain in childhood cancer survivors: A protocol for a single-center, pilot randomized controlled trial. Contemporary Clinical Trials Communications, 2023. 36. | Paediatric Population |
| 28 | Daviu Cobián, C., et al., Impact of FRAilty screening and Geriatric assessment and INtervention in older patients with epithelial Ovarian Cancer: A multicenter randomized clinical trial protocol (FRAGINOC). Journal of Geriatric Oncology, 2024. 15(3). | Wrong Intervention |
| 29 | Dennett, A.M., et al., Efficacy of Group Exercise-Based Cancer Rehabilitation Delivered via Telehealth (TeleCaRe): Protocol for a Randomized Controlled Trial. JMIR Research Protocols, 2022. 11(7): p. e38553. | Wrong Intervention |
| 30 | Dennett, A.M., et al., Prehabilitation to improve outcomes afteR Autologous sTem cEll transplantation (PIRATE): A pilot randomised controlled trial protocol. Plos one, 2023. 18(4 April | Wrong Intervention |
| 31 | DeNysschen, C.A., et al., Nutritional symptom and body composition outcomes of aerobic exercise in women with breast cancer. Clinical Nursing Research, 2011. 20(1): p. 29-46. | Not Protocol Paper |
| 32 | Dhillon, H.M., et al., The impact of physical activity on fatigue and quality of life in lung cancer patients: A randomised controlled trial protocol. BMC cancer, 2012. 12. | Wrong Intervention |
| 33 | Edbrooke, L., et al., Benefits of home-based multidisciplinary exercise and supportive care in inoperable non-small cell lung cancer - protocol for a phase II randomised controlled trial. BMC cancer, 2017. 17(1). | Wrong Intervention |
| 34 | El-Khoury, F., et al., Effectiveness of a community-based multicomponent lifestyle intervention (the ADA programme) to improve the quality of life of French breast cancer survivors: Protocol for a pragmatic cluster randomised trial and embedded qualitative study. BMJ open, 2024. 14(3). | Wrong Intervention |
| 35 | Ester, M., et al., Protocol: A cluster randomized controlled trial of a mobile application to support physical activity maintenance after an exercise oncology program. Contemporary clinical trials, 2021. 107. | Wrong Intervention |
| 36 | Evans, H., et al., A tailored eHealth guidance and exercise prescription tool for men with metastatic prostate cancer: A protocol for feasibility, safety and usability testing. BJU International, 2018. 122: p. 19. | Wrong Intervention |
| 37 | Evans, H.E.L., et al., Evaluating a web- and telephone-based personalised exercise intervention for individuals living with metastatic prostate cancer (ExerciseGuide): protocol for a pilot randomised controlled trial. Pilot and Feasibility Studies, 2021. 7(1). | Wrong Intervention |
| 38 | Falz, R., et al., CRBP-TS - evaluation of a home-based training and health care program for colorectal, breast, and prostate cancer using telemonitoring and self-management: study protocol for a randomized controlled trial. BMC Sports Science, Medicine and Rehabilitation, 2021. 13(1). | Wrong Intervention |
| 39 | Feder, K.M., et al., Effectiveness of an expert assessment and individualised treatment compared with a minimal home-based exercise program in women with late-term shoulder impairments after primary breast cancer surgery: study protocol for a randomised controlled trial. Trials, 2022. 23(1). | Wrong Intervention |
| 40 | Fernández-Rodríguez, E.J., et al., Specific autonomy recovery programme in a comprehensive rehabilitation on functionality and respiratory parameters in oncological patients with dyspnoea. Study protocol. BMC nursing, 2021. 20(1). | Wrong Intervention |
| 41 | Fernández-Rodriguez, E.J., et al., Multimodal physical exercise and functional rehabilitation program in oncological patients with asthenia. study protocol. BMC nursing, 2021. 20(1). | Wrong Intervention |
| 42 | Focht, B.C., et al., The Individualized Diet and Exercise Adherence Pilot Trial (IDEA-P) in prostate cancer patients undergoing androgen deprivation therapy: Study protocol for a randomized controlled trial. Trials, 2014. 15(1). | Wrong Intervention |
| 43 | Frensham, L.J., et al., Steps toward improving diet and exercise for cancer survivors (STRIDE): A quasi-randomised controlled trial protocol. BMC cancer, 2014. 14(1). | Wrong Intervention |
| 44 | Fugazzaro, S., et al., PUREAIR protocol: Randomized controlled trial of intensive pulmonary rehabilitation versus standard care in patients undergoing surgical resection for lung cancer. BMC cancer, 2017. 17(1). | Wrong Intervention |
| 45 | Galiano-Castillo, N., et al., Telehealth system (e-CUIDATE) to improve quality of life in breast cancer survivors: Rationale and study protocol for a randomized clinical trial. Trials, 2013. 14(1). | Wrong Intervention |
| 46 | Gallyer, V., et al., Getting Recovery Right after Neck Dissection (GRRAND-F): Mixed-methods feasibility study to design a pragmatic randomised controlled trial protocol. BMJ open, 2021. 11(6). | Wrong Intervention |
| 47 | Galvão, D.A., et al., Weight loss for overweight and obese patients with prostate cancer: a study protocol of a randomised trial comparing clinic-based versus Telehealth delivered EXercise and nutrition intervention (the TelEX trial). BMJ open, 2022. 12(6). | Wrong Intervention |
| 48 | Gil-Cosano, J.J., et al., The effect of an online exercise programme on bone health in paediatric cancer survivors (iBoneFIT): study protocol of a multi-centre randomized controlled trial. BMC Public Health, 2020. 20(1): p. 1520 | Paediatric Population |
| 49 | Gnagnarella, P., et al., Promoting weight loss through diet and exercise in overweight or obese breast cancer survivors (InForma): Study protocol for a randomized controlled trial. Trials, 2016. 17(1). | Wrong Intervention |
| 50 | Götte, M., et al., A physical activity program on cardiorespiratory fitness in children and adolescents following acute cancer treatment (POWER): study protocol for a randomized controlled trial. Oncology Research and Treatment, 2022. 45: p. 194. | Paediatric Population |
| 51 | Granger, C.L., et al., Effect of a postoperative home-based exercise and self-management programme on physical function in people with lung cancer (CAPACITY): Protocol for a randomised controlled trial. BMJ Open Respiratory Research, 2022. 9(1). | Wrong Intervention |
| 52 | Hackshaw-McGeagh, L., et al., Prostate cancer - evidence of exercise and nutrition trial (PrEvENT): Study protocol for a randomised controlled feasibility trial. Trials, 2016. 17(1). | Wrong Intervention |
| 53 | Hajdú, S.F., et al., Swallowing therapy and progressive resistance training in head and neck cancer patients undergoing radiotherapy treatment: randomized control trial protocol and preliminary data. Acta Oncologica, 2017. 56(2): p. 354-359. | Wrong Intervention |
| 54 | Hansen, A., et al., The effect of an interdisciplinary rehabilitation intervention comparing HRQoL, symptom burden and physical function among patients with primary glioma: An RCT study protocol. BMJ open, 2014. 4(10). | Wrong Intervention |
| 55 | Harris, J., et al., CanWalk: study protocol for a randomized feasibility trial of a walking intervention for people with recurrent or metastatic cancer. Pilot & Feasibility Studies, 2015. 1: p. 7. | Wrong Intervention |
| 56 | Hathiramani, S., et al., Relaxation and Exercise in Lymphoma Survivors (REIL Study): A Randomised Clinical Trial Protocol. JCO Global Oncology, 2018. 4. | Wrong Intervention |
| 57 | Hauth, F., et al., An Activity Tracker-Guided Physical Activity Program for Patients Undergoing Radiotherapy: Protocol for a Prospective Phase III Trial (OnkoFit I and II Trials). JMIR Research Protocols, 2021. 10(9). | Wrong Intervention |
| 58 | Henkin, J.S., et al., Telehealth multicomponent exercise and health education in breast cancer patients undergoing primary treatment: rationale and methodological protocol for a randomized clinical trial (ABRACE: Telehealth). Trials, 2023. 24(1). | Wrong Intervention |
| 59 | Hidde, M., et al., Take charge during treatment: A planned exercise protocol to evaluate disparities and cardiovascular outcomes in Black and White patients with breast cancer undergoing treatment. Journal of clinical oncology, 2022. 40(16). | Not Protocol Paper |
| 60 | Hirschey, R., et al., Protocol for Moving On: a randomized controlled trial to increase outcome expectations and exercise among breast cancer survivors. Nursing open, 2018. 5(1): p. 101-108. | Wrong Intervention |
| 61 | Ho, J.W., et al., Study protocol for "Moving Bright, Eating Smart"- A phase 2 clinical trial on the acceptability and feasibility of a diet and physical activity intervention to prevent recurrence in colorectal cancer survivors. BMC Public Health, 2013. 13: p. 487. | Wrong Intervention |
| 62 | Huang, C., et al., Effect of a family-involvement combined aerobic and resistance exercise protocol on cancer-related fatigue in patients with breast cancer during postoperative chemotherapy: Study protocol for a quasi-randomised controlled trial. BMJ open, 2023. 13(3) | Not Randomised Controlled Trial |
| 63 | Hunt, B.E., et al., EFFECT OF ENHANCED EXERCISE PROTOCOL ON FATIGUE & FUNCTION FOR PATIENTS UNDERGOING STEM CELL TRANSPLANT. Rehabilitation Oncology, 2023. 41(2): p. 104-105. | Not Protocol Paper |
| 64 | Hunter, H., et al., The Get Moving Trial : A phase I/II RCT of home-based (P)rehabilitation ((P)REHAB) with ExerciseRx in muscle-invasive bladder cancer (MIBC)-Study protocol for a randomized controlled trial. Journal of clinical oncology, 2024. 42(4). | Not Protocol Paper |
| 65 | Jaeger, A., et al., Study protocol of an exercise and nutrition intervention for ovarian cancer patients during and after first line chemotherapy-a randomized controlled trial. Geburtshilfe und Frauenheilkunde, 2018. 78(10). | Wrong Intervention |
| 66 | James, E.L., et al., Exercise and nutrition routine improving cancer health (ENRICH): the protocol for a randomized efficacy trial of a nutrition and physical activity program for adult cancer survivors and carers. BMC Public Health, 2011. 11: p. 236. | Wrong Intervention |
| 67 | Jansen, F., et al., Effectiveness and cost-utility of a guided self-help exercise program for patients treated with total laryngectomy: Protocol of a multi-center randomized controlled trial. BMC cancer, 2016. 16(1). | Wrong Intervention |
| 68 | Jensen, W., et al., Effects of exercise training programs on physical performance and quality of life in patients with metastatic lung cancer undergoing palliative chemotherapy-A study protocol. Contemporary clinical trials, 2014. 37(1): p. 120-128. | Protocol Published Pre-SPIRIT Checklist |
| 69 | Keen, C., et al., Is it feasible to conduct a randomised controlled trial of pretransplant exercise (prehabilitation) for patients with multiple myeloma awaiting autologous haematopoietic stem cell transplantation? Protocol for the PREeMPT study. BMJ open, 2018. 8(3). | Not Randomised Controlled Trial |
| 70 | Kennedy, F., et al., Promoting physical activity via a smartphone application in people living with breast, prostate or colorectal cancer: study protocol for a randomised pilot trial (APPROACH). Psycho-Oncology, 2022. 31: p. 20-20. | Wrong Intervention |
| 71 | Kilbreath, S.L., et al., Progressive resistance training and stretching following surgery for breast cancer: Study protocol for a randomised controlled trial. BMC cancer, 2006. 6. | Protocol Published Pre-SPIRIT Checklist |
| 72 | Kim, I., et al., Effectiveness of a personalized digital exercise and nutrition-based rehab program for patients with gastric cancer after surgery: Study protocol for a randomized controlled trial. Digital health, 2023. 9: p. 20552076231187602. | Wrong Intervention |
| 73 | Kjeldsted, E., et al., Neo-train: study protocol and feasibility results for a two-arm randomized controlled trial investigating the effect of supervised exercise during neoadjuvant chemotherapy on tumour response in patients with breast cancer. BMC cancer, 2023. 23(1). | Wrong Intervention |
| 74 | Kohler, B.E., et al., Physical ACTivity in Survivorship (PACTS): study protocol for a randomized controlled trial evaluating a goal-directed therapeutic exercise program in pediatric posterior fossa brain tumor survivors. BMC Pediatrics, 2021. 21(1). | Paediatric Population |
| 75 | Kriellaars, Y., et al., The effect of physical exercise during radiotherapy on physical performance in patients with head and neck cancer: a trial within cohorts study protocol, the vital study. BMC cancer, 2024. 24(1). | Not Randomised Controlled Trial |
| 76 | Krok-Schoen, J.L., et al., E-PROOF: E-intervention for protein intake and resistance training to optimize function: A study protocol. Plos one, 2024. 19(5 May). | Wrong Intervention |
| 77 | Lambert, S.D., et al., A study protocol for a multicenter randomized pilot trial of a dyadic, tailored, web-based, psychosocial, and physical activity self-management program (TEMPO) for men with prostate cancer and their caregivers. Pilot and Feasibility Studies, 2021. 7(1). | Wrong Intervention |
| 78 | Langballe, R., et al., NAVIGATE: improving survival in vulnerable patients with lung cancer through nurse navigation, symptom monitoring and exercise - study protocol for a multicentre randomised controlled trial. BMJ open, 2022. 12(10). | Wrong Intervention |
| 79 | Lantis, K., et al., Biomechanical effect of neurologic dance training (NDT) for breast cancer survivors with chemotherapy-induced neuropathy: study protocol for a randomized controlled trial and preliminary baseline data. Trials, 2023. 24(1). | Duplicate |
| 80 | Lantis, K.D., et al., Biomechanical effect of neurologic dance training (NDT) for breast cancer survivors with chemotherapy-induced neuropathy: study protocol for a randomized controlled trail and preliminary baseline data. Research square, 2023 | Wrong Intervention |
| 81 | Lee, K., et al., <i>T</i>elehealth exercise to <i>I</i>mprove <i>P</i>hysical function and frailty in patients with multiple myeloma treated with autologous hematopoietic Stem cell transplantation (TIPS): protocol of a randomized controlled trial. Trials, 2022. 23(1). | Duplicate |
| 82 | Li, C., et al., The effects of qigong intervention based on the Internet on quality of life and physical fitness in Chinese postoperative breast cancer patients: a protocol of randomized controlled trial. Trials, 2023. 24(1). | Wrong Intervention |
| 83 | Liao, Y., et al., Using wearable biological sensors to provide personalized feedback to motivate behavioral changes: Study protocol for a randomized controlled physical activity intervention in cancer survivors (Project KNOWN). Plos one, 2022. 17(9 September). | Wrong Intervention |
| 84 | Little, R.B., et al., <u>Ro</u>le of Gut <u>M</u>icrobe Composition in Psychosocial Symptom Response to <u>E</u>xercise Training in Breast Cancer Survivors (ROME) study: protocol for a randomised controlled trial. BMJ open, 2024. 14(5): p. e081660. | Duplicate |
| 85 | Liu, X.Y., et al., Comparative effects of Yi Jin Jing versus Tai Chi exercise training on benign prostatic hyperplasia-related outcomes in older adults: study protocol for a randomized controlled trial. Trials, 2016. 17. | Wrong Intervention |
| 86 | Liu, L., et al., An integrative Tai Chi program for patients with breast cancer undergoing cancer therapy: study protocol for a randomized controlled feasibility study. Journal of Integrative Medicine, 2018. 16(2): p. 99-105. | Wrong Intervention |
| 87 | Liu, Y., et al., The protocol for the prehabilitation for thoracic surgery study: a randomized pragmatic trial comparing a short home-based multimodal program to aerobic training in patients undergoing video-assisted thoracoscopic surgery lobectomy. Trials, 2023. 24(1). | Wrong Intervention |
| 88 | Lopez-Garzon, M., et al., Prevention of Chemotherapy-Induced Peripheral Neuropathy With PRESIONA, a Therapeutic Exercise and Blood Flow Restriction Program: A Randomized Controlled Study Protocol. Physical Therapy, 2022. 102(3). | Wrong Intervention |
| 89 | Macías-Valle, A., et al., Exercise effects on functional capacity and quality of life in older patients with colorectal cancer: study protocol for the ECOOL randomized controlled trial. BMC geriatrics, 2023. 23(1): p. 314. | Wrong Intervention |
| 90 | Martinez Aguirre-Betolaza, A., J. Cacicedo, and A. Castaneda-Babarro, Creatine Supplementation and Resistance Training in Patients With Breast Cancer (CaRTiC Study): protocol for a Randomized Controlled Trial. American journal of clinical oncology, 2023. | Duplicate |
| 91 | Maxwell-Smith, C., et al., Wearable Activity Technology And Action-Planning (WATAAP) to promote physical activity in cancer survivors: Randomised controlled trial protocol. International Journal of Clinical & Health Psychology, 2018. 18(2): p. 124-132. | Wrong Intervention |
| 92 | McCloy, K., et al., Evaluating the effectiveness of mindfulness alone compared to exercise and mindfulness on fatigue in women with gynaecology cancer (GEMS): Protocol for a randomised feasibility trial. 2022. | Wrong Intervention |
| 93 | McCloy, K., et al., Evaluating the effectiveness of mindfulness alone compared to exercise and mindfulness on fatigue in women with gynaecology cancer (GEMS): Protocol for a randomised feasibility trial. Plos one, 2023. 18(10 October). | Wrong Intervention |
| 94 | McGeagh, L., et al., Prostate cancer–Exercise and Metformin Trial (Pre-EMpT): study protocol for a feasibility factorial randomized controlled trial in men with localised or locally advanced prostate cancer. Pilot and Feasibility Studies, 2022. 8(1). | Wrong Intervention |
| 95 | McNaught, E., et al., Supported exercise TrAining for Men wIth prostate caNcer on Androgen deprivation therapy (STAMINA): study protocol for a randomised controlled trial of the clinical and cost-effectiveness of the STAMINA lifestyle intervention compared with optimised usual care, including internal pilot and parallel process evaluation. Trials, 2024. 25(1). | Wrong Intervention |
| 96 | McNaught, E., et al., <i>S</i>upported exercise <i>T</i>r<i>A</i>ining for <i>M</i>en w<i>I</i>th prostate ca<i>N</i>cer on <i>A</i>ndrogen deprivation therapy (STAMINA): study protocol for a randomised controlled trial of the clinical and cost-effectiveness of the STAMINA lifestyle intervention compared with optimised usual care, including internal pilot and parallel process evaluation. Trials, 2024. 25(1). | Duplicate |
| 97 | Meier-Girard, D., et al., Eurythmy therapy versus slow movement fitness in the treatment of fatigue in metastatic breast cancer patients: Study protocol for a randomized controlled trial. Trials, 2020. 21(1). | Wrong Intervention |
| 98 | Mikkelsen, M.K., et al., Engaging the older cancer patient; Patient Activation through Counseling, Exercise and Mobilization - Pancreatic, Biliary tract and Lung cancer (PACE-Mobil-PBL) - Study protocol of a randomized controlled trial. BMC cancer, 2018. 18(1). | Wrong Intervention |
| 99 | Milbury, K., et al., A research protocol for a pilot, randomized controlled trial designed to examine the feasibility of a dyadic versus individual yoga program for family caregivers of glioma patients undergoing radiotherapy. Pilot and Feasibility Studies, 2019. 5(1). | Wrong Intervention |
| 100 | Moyen, A., et al., A Novel Multimodal Intervention for Surgical Prehabilitation on Functional Recovery and Muscle Characteristics in Patients With Lung Cancer – A Study Protocol. Current Developments in Nutrition, 2023. 7. | Not Protocol Paper |
| 101 | Munsie, C., et al., A supervised exercise intervention during cancer treatment for adolescents and young adults—FiGHTING F!T: study protocol of a randomised controlled trial. Trials, 2021. 22(1). | Paediatric Population |
| 102 | Murphy, K., et al., A comparison of aerobic- and resistance-emphasised exercise on cardiometabolic health and quality of life in men receiving androgen deprivation therapy for prostate cancer: protocol for a feasibility trial. Contemporary clinical trials, 2023. 136: p. 107388. | Duplicate |
| 103 | Natalucci, V., et al., Movement and health beyond care, MoviS: study protocol for a randomized clinical trial on nutrition and exercise educational programs for breast cancer survivors. Trials, 2023. 24(1). | Wrong Intervention |
| 104 | Nevo, E.O., et al., Digitally distributed Yoga Intervention in Breast Cancer Rehabilitation (DigiYoga CaRe): protocol for a randomised controlled trial. BMJ open, 2022. 12(11). | Wrong Intervention |
| 105 | Nilsen, T.S., et al., Correction: Effects of Aerobic Exercise on Cardiorespiratory Fitness, Cardiovascular Risk Factors, and Patient-Reported Outcomes in Long-Term Breast Cancer Survivors: Protocol for a Randomized Controlled Trial. JMIR Research Protocols, 2023. 12: p. e54462. | Not Protocol Paper |
| 106 | Nishiyama, N., et al., The efficacy of specialised rehabilitation using the Op-reha Guide for cancer patients in palliative care units: protocol of a multicentre, randomised controlled trial (JORTC-RHB02). BMC Palliative Care, 2020. 19(1). | Wrong Intervention |
| 107 | Nissim, M., et al., Effects of aquatic exercise program versus on-land exercise program on cancer-related fatigue, neuropathy, activity and participation, quality of life, and return to work for cancer patients: study protocol for a randomized controlled trial. BMC Complementary Medicine and Therapies, 2024. 24(1). | Wrong Intervention |
| 108 | Noh, H., et al., Impact of a one-year supervised physical activity program on long-term cancer-related fatigue and mediating effects of the gut microbiota in metastatic testicular cancer patients: protocol of the prospective multicentre, randomized controlled phase-III STARTER trial. BMC cancer, 2024. 24(1). | Wrong Intervention |
| 109 | Oei, S.L., et al., TANGO: effect of tango Argentino on cancer-associated fatigue in breast cancer patients—study protocol for a randomized controlled trial. Trials, 2021. 22(1). | Wrong Intervention |
| 110 | Oei, S.L., et al., Study Protocol of a Randomized Controlled Trial: Argentine Tango for Breast Cancer Patients with Fatigue. Oncology Research and Treatment, 2022. 45: p. 215. | Wrong Intervention |
| 111 | O'Neill, L., et al., Rehabilitation strategies following oesophagogastric and Hepatopancreaticobiliary cancer (ReStOre II): A protocol for a randomized controlled trial. BMC cancer, 2020. 20(1). Wrong Intervention |  |
| 112 | Onerup, A., et al., The effect of pre- and post-operative physical activity on recovery after colorectal cancer surgery (PHYSSURG-C): Study protocol for a randomised controlled trial. Trials, 2017. 18(1). | Wrong Intervention |
| 113 | Onerup, A., et al., Correction to: The effect of pre- and post-operative physical activity on recovery after colorectal cancer surgery (PHYSSURG-C): study protocol for a randomised controlled trial (Trials, (2017), 18, 1, (212), 10.1186/s13063-017-1949-9). Trials, 2020. 21(1). | Not Protocol Paper |
| 114 | Onerup, A., et al., Correction to: The effect of pre- and post-operative physical activity on recovery after colorectal cancer surgery (PHYSSURG-C): study protocol for a randomised controlled trial. Trials [electronic resource], 2020. 21(1): p. 1030. | Not Protocol Paper |
| 115 | Onerup, A., et al., The effect of pre- and post-operative physical activity on recovery after colorectal cancer surgery (PHYSSURG-C): study protocol for a randomised controlled trial (vol 18, 212, 2017). Trials, 2020. 21(1). | Not Protocol Paper |
| 116 | Owen, P.J., et al., Efficacy of a multi-component exercise programme and nutritional supplementation on musculoskeletal health in men treated with androgen deprivation therapy for prostate cancer (IMPACT): Study protocol of a randomised controlled trial. Trials, 2017. 18(1). | Wrong Intervention |
| 117 | Pan, H., et al., Tai Chi Chuan in postsurgical non-small cell lung cancer patients: Study protocol for a randomized controlled trial. Trials, 2018. 19(1). | Wrong Intervention |
| 118 | Pekmezi, D., et al., Adapting MultiPLe behavior Interventions that eFfectively Improve (AMPLIFI) cancer survivor health: program project protocols for remote lifestyle intervention and assessment in 3 inter-related randomized controlled trials among survivors of obesity-related cancers. BMC cancer, 2022. 22(1). | Wrong Intervention |
| 119 | Phillips, S., et al., A Technology-Based Physical Activity Intervention for Patients With Metastatic Breast Cancer (Fit2ThriveMB): Protocol for a Randomized Controlled Trial. JMIR Research Protocols, 2021. 10(4): p. e24254. | Wrong Intervention |
| 120 | Piper, K.S., et al., The SaVe project – Sarcopenia and Vertigo in aging patients with colorectal cancer: A study protocol for three randomized controlled trials. Journal of Geriatric Oncology, 2024. 15(4). | Wrong Intervention |
| 121 | Potthoff, K., et al., Randomized controlled trial to evaluate the effects of progressive resistance training compared to progressive muscle relaxation in breast cancer patients undergoing adjuvant radiotherapy: the BEST study. BMC cancer, 2013. 13(1): p. 162-162. | Protocol Published Pre-SPIRIT Checklist |
| 122 | Price, J. and J. Brunet, A single-subject research design evaluating a co-created yoga program for adults with gynecologic cancer: feasibility study protocol. Pilot and Feasibility Studies, 2024. 10(1). | Not Randomised Controlled Trial |
| 123 | Qiu, L., et al., Comparison of the effects of different functional exercise sequences on lymphedema in breast cancer: Protocol for an exploratory randomised controlled cross-over trial. BMJ open, 2024. 14(3). | Wrong Intervention |
| 124 | Quist, M., et al., "EXHALE": exercise as a strategy for rehabilitation in advanced stage lung cancer patients: a randomized clinical trial comparing the effects of 12 weeks supervised exercise intervention versus usual care for advanced stage lung cancer patients. BMC cancer, 2013. 13(1): p. 1-14. | Protocol Published Pre-SPIRIT 2013 Checklist |
| 125 | Ribeiro, C., et al., Feasibility of Home Vs. Hospital based resistance training for advanced cancer patients: Study protocol of a phase II trial. Supportive Care in Cancer, 2016. 24(1): p. S215-S216. | Not Protocol Paper |
| 126 | Richter, S., et al., Progressive postresection program (pPRP) after pancreatic resection: Study protocol for a randomized controlled trial. Trials, 2016. 17(1). | Wrong Intervention |
| 127 | Ritvo, P., et al., Smartphone-Enabled Health Coaching Intervention (iMOVE) to Promote Long-Term Maintenance of Physical Activity in Breast Cancer Survivors: Protocol for a Feasibility Pilot Randomized Controlled Trial. JMIR Research Protocols, 2017. 6(8): p. e165. | Wrong Intervention |
| 128 | Roy, B.L., et al., Effect of prehabilitation in gastro-oesophageal adenocarcinoma: Study protocol of a multicentric, randomised, control trial-the PREHAB study. BMJ open, 2016. 6(12). | Wrong Intervention |
| 129 | Sattar, S., et al., Testing the feasibility and effects of the virtual stable program on reducing fall risk among community-dwelling older adults with cancer: Protocol for a randomized controlled trial. Supportive Care in Cancer, 2021. 29(SUPPL 1): p. S72. | Not Protocol Paper |
| 130 | Sattar, S., et al., Feasibility and efficacy of a remotely delivered fall prevention exercise program for community-dwelling older adults with cancer: Protocol for the STABLE trial. Journal of Geriatric Oncology, 2022. 13(8): p. 1273-1280. | Wrong Intervention |
| 131 | Saxton, J.M., et al., Study protocol to investigate the effect of a lifestyle intervention on body weight, psychological health status and risk factors associated with disease recurrence in women recovering from breast cancer treatment. BMC cancer, 2006. 6: p. 35. | Wrong Intervention |
| 132 | Saxton, J.M., et al., Study protocol to investigate the effect of a lifestyle intervention on body weight, psychological health status and risk factors associated with disease recurrence in women recovering from breast cancer treatment [ISRCTN08045231]. BMC cancer, 2006. 6. | Wrong Intervention |
| 133 | Schega, L., et al., Evaluation of a supervised multi-modal physical exercise program for prostate cancer survivors in the rehabilitation phase: Rationale and study protocol of the ProCaLife study. Contemporary clinical trials, 2015. 45. | Not Randomised Controlled Trial |
| 134 | Schenk, A., et al., Influence of a single exercise session on natural killer cell cytotoxicity and tumor infiltration in preoperative esophageal carcinoma patients-a study protocol. Oncology Research and Treatment, 2016. 39: p. 153. | Wrong Intervention |
| 135 | Schmidt-Andersen, P., et al., Integrative Neuromuscular Training in Adolescents and Children Treated for Cancer (INTERACT): Study Protocol for a Multicenter, Two-Arm Parallel-Group Randomized Controlled Superiority Trial. Frontiers in Pediatrics, 2022. 10. | Paediatric Population |
| 136 | Schumann, M., et al., Feasibility of high-intensity interval training with hyperoxia vs. intermittent hyperoxia and hypoxia in cancer patients undergoing chemotherapy – Study protocol of a randomized controlled trial. Contemporary Clinical Trials Communications, 2017. 8: p. 213-217. | Wrong Intervention |
| 137 | Scott, E., et al., The effect of a lifestyle intervention on body weight, psychological health status, and risk factors associated with disease recurrence in women recovering from breast cancer treatment: Study protocol and interim findings. Journal of Nutrition, 2007. 137(1): p. 284S-284S. | Wrong Intervention |
| 138 | Short, C.E., et al., Move more for life: The protocol for a randomised efficacy trial of a tailored-print physical activity intervention for post-treatment breast cancer survivors. BMC cancer, 2012. 12. | Wrong Intervention |
| 139 | Smith, J. and J. Richardson, Bridging the gap: Incorporating exercise evidence into clinical practice in breast cancer care-a study protocol. Archives of Physical Medicine and Rehabilitation, 2017. 98(10): p. e38. | Not Protocol Paper |
| 140 | Smith-Turchyn, J., et al., Evaluation of a novel strategy to implement exercise evidence into clinical practice in breast cancer care: protocol for the NEXT-BRCA randomised controlled trial. BMJ open sport & exercise medicine, 2020. 6(1): p. e000922. | Wrong Intervention |
| 141 | Smith-Turchyn, J., et al., Connecting breast cancer survivors for exercise: protocol for a two-arm randomized controlled trial. BMC Sports Science, Medicine and Rehabilitation, 2021. 13(1). | Wrong Intervention |
| 142 | Sohl, S.J., et al., Intervention Protocol for Investigating Yoga Implemented During Chemotherapy. International journal of yoga therapy, 2016. 26(1): p. 103-111. | Wrong Intervention |
| 143 | Soriano-Maldonado, A., A. Carrera-Ruiz, and D.M. Díez-Fernández, Effects of a 12-week resistance and aerobic exercise program on muscular strength and quality of life in breast cancer survivors: Study protocol for the EFICAN randomized controlled trial (vol 98, e17625, 2019). Medicine, 2019. 98(49). | Not Protocol Paper |
| 144 | Spence, R.R., et al., Randomised controlled trial of a supervised exercise rehabilitation program for colorectal cancer survivors immediately after chemotherapy: Study protocol. BMC cancer, 2007. 7 | Protocol Published Pre-SPIRIT 2013 Checklist |
| 145 | Steffens, D., et al., PRehabllitatiOn with pReoperatIve exercise and educaTion for patients undergoing major abdominal cancer surgerY: protocol for a multicentre randomised controlled TRIAL (PRIORITY TRIAL). BMC cancer, 2022. 22(1). | Wrong Intervention |
| 146 | Stelten, S., et al., Rationale and study protocol of the Physical Activity and Dietary intervention in women with OVArian cancer (PADOVA) study: A randomised controlled trial to evaluate effectiveness of a tailored exercise and dietary intervention on body composition, physical function and fatigue in women with ovarian cancer undergoing chemotherapy. BMJ open, 2020. 10(11) | Wrong Intervention |
| 147 | Streckmann, F., et al., Individually tailored whole-body vibration training to reduce symptoms of chemotherapy-induced peripheral neuropathy: Study protocol of a randomised controlled trial-VANISH. BMJ open, 2019. 9(4). | Wrong Intervention |
| 148 | Sun, Y., et al., Multimodal prehabilitation to improve the clinical outcomes of frail elderly patients with gastric cancer: a study protocol for a multicentre randomised controlled trial (GISSG<sup>+</sup>2201). BMJ open, 2023. 13(10): p. e071714. | Wrong Intervention |
| 149 | Sun, Y., et al., Multimodal prehabilitation to improve the clinical outcomes of frail elderly patients with gastric cancer: a study protocol for a multicentre randomised controlled trial (GISSG + 2201). BMJ open, 2023. 13(10). | Wrong Intervention |
| 150 | Teran-Wodzinski, P., et al., Assessing gait, balance, and muscle strength among breast cancer survivors with chemotherapy-induced peripheral neuropathy (CIPN): study protocol for a randomized controlled clinical trial. Trials, 2022. 23(1). | Wrong Intervention |
| 151 | Teran-Wodzinski, P., et al., Effects of home-based exercise intervention in breast cancer survivors with chemotherapy-induced peripheral neuropathy: Study protocol. Rehabilitation Oncology, 2021. 39(2): p. E25. | Not Protocol Paper |
| 152 | Tønnesen, H., et al., STRONG for Surgery & Strong for Life — against all odds: intensive prehabilitation including smoking, nutrition, alcohol and physical activity for risk reduction in cancer surgery — a protocol for an RCT with nested interview study (STRONG-Cancer). Trials, 2022. 23(1). | Wrong Intervention |
| 153 | Tsuji, K., et al., Effect of home-based high-intensity interval training and behavioural modification using information and communication technology on cardiorespiratory fitness and exercise habits among sedentary breast cancer survivors: Habit-B study protocol for a randomised controlled trial. BMJ open, 2019. 9(8). | Wrong Intervention |
| 154 | Umin, Effect of home-based high intensity intermittent training and behavioral modification using information and communication technology on cardiorespiratory fitness and exercise habits among sedentary breast cancer survivors: habit-B study protocol for a randomized controlled trial. https://trialsearch.who.int/Trial2.aspx?TrialID=JPRN-UMIN000036400, 2019. | Wrong Intervention |
| 155 | Uth, J., et al., Effects of recreational soccer in men with prostate cancer undergoing androgen deprivation therapy: Study protocol for the 'FC Prostate' randomized controlled trial. BMC cancer, 2013. 13. | Wrong Intervention |
| 156 | Van der Gucht, K., et al., A mindfulness-based intervention for breast cancer patients with cognitive impairment after chemotherapy: study protocol of a three-group randomized controlled trial. Trials [electronic resource], 2020. 21(1): p. 290. | Wrong Intervention |
| 157 | Vear, N., et al., Effects of exercise on sexual function and cardiovascular health in menwith prostate cancer (ESCA): A Study Protocol. Asia-Pacific Journal of Clinical Oncology, 2019. 15: p. 66-67. | Not Protocol Paper |
| 158 | Wagoner, C.W., et al., Multiphasic exercise prehabilitation for patients undergoing surgery for head and neck cancer: a hybrid effectiveness-implementation study protocol. Supportive Care in Cancer, 2023. 31(12). | Not Randomised Controlled Trial |
| 159 | Wang, C.C., et al., Effects of a nurse-led Tai Chi programme on improving quality of life, mental wellbeing, and physical function of women with breast cancer: Protocol for a randomized controlled trial. Women's Health, 2022. 18. | Wrong Intervention |
| 160 | Wang, J., et al., Quality of life between home-based and outpatient pulmonary rehabilitation in patients after surgical resection for lung cancer: Protocol for a prospective, single-blind, randomised controlled trial. BMJ open, 2023. 13(5) | Wrong Intervention |
| 161 | Webb, J., et al., The Effect of the Move More Pack on the Physical Activity of Cancer Survivors: protocol for a Randomized Waiting List Control Trial with Process Evaluation. JMIR Research Protocols, 2017. 6(11): p. e220. | Wrong Intervention |
| 162 | Wei, X.L., et al., Effect of Baduanjin exercise intervention on cognitive function and quality of life in women with breast cancer receiving chemotherapy: study protocol of a randomized controlled trial. Trials, 2021. 22(1). | Wrong Intervention |
| 163 | Welte, S.E., et al., Differentiated resistance training of the paravertebral muscles in patients with unstable spinal bone metastasis under concomitant radiotherapy: Study protocol for a randomized pilot trial. Trials, 2017. 18(1). | Wrong Intervention |
| 164 | West, M., et al., The Wessex Fit-4-Cancer Surgery Trial (WesFit): a protocol for a factorial-design, pragmatic randomised-controlled trial investigating the effects of a multi-modal prehabilitation programme in patients undergoing elective major intra-cavity cancer surgery. F1000Research, 2021. 10: p. 952 | Duplicate |
| 165 | Wijma, A.G., et al., Cardiac and intramuscular adaptations following short-term exercise prehabilitation in unfit patients scheduled to undergo hepatic or pancreatic surgery: Study protocol of a multinuclear MRI study. BMJ Open Gastroenterology, 2023. 10(1). | Not Randomised Controlled Trial |
| 166 | Winters-Stone, K.M., et al., The effect of resistance training on muscle strength and physical function in older, postmenopausal breast cancer survivors: a randomized controlled trial. J Cancer Surviv, 2012. 6(2): p. 189-99. | Protocol Published Pre-SPIRIT 2013 Checklist |
| 167 | Wong, S.G., et al., Evaluation of a physiatrist-directed prehabilitation intervention in frail patients with colorectal cancer: A randomised pilot study protocol. BMJ open, 2017. 7(6). | Wrong Intervention |
| 168 | Xiaosheng, D., et al., The effects of combined exercise intervention based on Internet and social media software for postoperative patients with breast cancer: Study protocol for a randomized controlled trial. Trials, 2018. 19(1). | Wrong Intervention |
| 169 | Xu, H., et al., CIMmH, an mHealth Comprehensive Intervention Model for esophageal cancer patients: A Multi-center RCT Protocol. International Journal of Epidemiology, 2021. 50: p. i255. | Wrong Intervention |
| 170 | Yan, H., et al., Testing Home-based Exercise Strategies in Underserved Minority Cancer Patients Undergoing Chemotherapy (THRIVE) Trial: A study protocol. Cancer Epidemiology Biomarkers and Prevention, 2023. 32(1). | Not Protocol Paper |
| 171 | Yao, J., et al., The effect of comprehensive rehabilitation program plus chemotherapy on quality of life in patients with postoperative non-small-cell lung cancer: Study protocol of a multicenter randomized clinical trial. Trials, 2020. 21(1). | Wrong Intervention |
| 172 | Yao, L.Q., et al., Development and validation of a Tai chi intervention protocol for managing the fatigue-sleep disturbance-depression symptom cluster in female breast cancer patients. Complementary Therapies in Medicine, 2021. 56. | Wrong Intervention |
| 173 | Yao, L.Q., et al., Feasibility and potential effects of tai chi for the fatigue-sleep disturbance-depression symptom cluster in patients with breast cancer: Protocol of a preliminary randomised controlled trial. BMJ open, 2021. 11(8). | Wrong Intervention |
| 174 | Yeo, S.M., et al., The effect of mHealth-based exercise on Insulin Sensitivity for patients with Hepatocellular carcinoma and insulin resistance (mISH): protocol of a randomized controlled trial. Trials, 2022. 23(1). | Wrong Intervention |
| 175 | Yeo, S.M., et al., The effect of <i>m</i>Health-based exercise on <i>I</i>nsulin <i>S</i>ensitivity for patients with <i>H</i>epatocellular carcinoma and insulin resistance (mISH): protocol of a randomized controlled trial. Trials, 2022. 23(1). | Duplicate |
| 176 | Yuan, R.Z., et al., Effects of free range-of-motion upper limb exercise based on mirror therapy on shoulder function in patients after breast cancer surgery: study protocol for a randomized controlled trial. Trials, 2021. 22(1). | Wrong Intervention |
| 177 | Zakharenko, A.A., et al., Short-term prehabilitation of patients with colorectal cancer — protocol of a randomized trial. Koloproktologia, 2023. 22(2): p. 70-78. | Not Available in English |
| 178 | Zetzl, T., et al., Yoga intervention and reminder e-mails for reducing cancer-related fatigue - a study protocol of a randomized controlled trial. BMC psychology, 2019. 7(1): p. 64. | Wrong Intervention |
| 179 | Zheng, Y., et al., Does a pulmonary rehabilitation based ERAS program (PREP) affect pulmonary complication incidence, pulmonary function and quality of life after lung cancer surgery? Study protocol for a multicenter randomized controlled trial. BMC Pulmonary Medicine, 2020. 20(1). | Wrong Intervention |
| 180 | Zopf, E.M., et al., Implementation and scientific evaluation of rehabilitative sports groups for prostate cancer patients: study protocol of the ProRehab Study. BMC cancer, 2012. 12. | Not Randomised Controlled Trial |
| 181 | Anonymous, SupPoRtive Exercise Programmes for Accelerating REcovery after major ABdominal Cancer surgery trial (PREPARE-ABC): Study protocol for a multicentre randomized controlled trial. Colorectal Disease, 2021. 23(10): p. 2750-2760. | Duplicate |
| 182 | Anonymous, Effects of a 12-week resistance and aerobic exercise program on muscular strength and quality of life in breast cancer survivors: Study protocol for the EFICAN randomized controlled trial: Erratum. Medicine, 2019. 98(49): p. e18419. | Not Protocol Paper |
| 183 | Ctri, A study to evaluate the effectiveness of exercise protocol in decreasing pain,numbness and swelling in arm,improving range of motion and strength in arm and improving quality of life among women with breast cancer who have undergone breast surgery. https://trialsearch.who.int/Trial2.aspx?TrialID=CTRI/2016/04/006897, 2016. | Trial Registration Only |
| 184 | ChiCtr, Effects of Baduanjin exercise on cancer-related fatigue in patients with prostate cancer treated with androgen deprivation therapy: study protocol for a randomized controlled trial. https://trialsearch.who.int/Trial2.aspx?TrialID=ChiCTR2300074293, 2023. | Trial Registration Only |
| 185 | Erratum: Effects of a 12-week resistance and aerobic exercise program on muscular strength and quality of life in breast cancer survivors: Study protocol for the EFICAN randomized controlled trial (Medicine (United States) 98:44 (e17625) DOI: 10.1097/MD.0000000000017625). Medicine (United States), 2019. 98(49). Not Protocol Paper | Not Protocol Paper |
| 186 | Nct, Effects of 6 Week Reduced Exertion High Intensity Interval Training Protocol in Patients With Prostate Cancer. https://clinicaltrials.gov/show/NCT03308734, 2017. | Trial Registration Only |
| 187 | Nct, Effect of Prehabilitation in Gastroesophageal Adenocarcinoma: study Protocol of a Multicentric, Randomised Control Trial. https://clinicaltrials.gov/show/NCT02780921, 2016. | Wrong Intervention |
| 188 | Nct, Support and Rehabilitation Protocol for Non Metastatic Breast Cancer Patients in Complete Remission After Chemotherapy. https://clinicaltrials.gov/show/NCT01563588, 2012. | Trial Registration Only |
