# Supplementary material for: Dissemination planning in exercise oncology trials—a systematic review of trial protocols
Source: Support Care Cancer. 2025 May 15;33(6):473. doi: 10.1007/s00520-025-09532-4 (PMC12078369; doi:10.1007/s00520-025-09532-4)
Supplement: Supplementary file 3 — Supplementary file C (DOCX 95 KB) [file 520_2025_9532_MOESM3_ESM.docx]

**Supplementary Material C – Demographic Details of Included Protocols**

*Title:* Dissemination Planning in Exercise Oncology Trials - A Systematic Review of Trial Protocols

*Authors*: Emily Smyth ^1,2^, Lydia Politi^3^, Emer Guinan^1,2^, David Mockler^4^, and Linda O’Neill^1,2,5^

*Affiliations*:

^1^ Discipline of Physiotherapy, Trinity College Dublin, Dublin, Ireland

^2^ Trinity St James's Cancer Institute, Dublin, Ireland

^3^ School of Biochemistry and Immunology, Trinity College Dublin, The University of Dublin, Dublin, Ireland

^4^ John Stearne Library, Trinity Centre for Health Sciences, St. James’s Hospital, Dublin, Ireland

^5^Clinical Research Centre, School of Medicine, University College Dublin, Dublin, Ireland

*Corresponding Author*: Dr Linda O’Neill

E-mail: [loneill4@tcd.ie](mailto:loneill4@tcd.ie)

**Supplementary Material C – Demographic Details of Included Protocols**

| **Author, Date** | **Country** | **Planned Sample Size** | **Cancer Types** | **Timepoint in Cancer Trajectory of Intervention** | **Number of Trial Arms** | **Exercise Intervention** | **Comparator 1** | **Comparator 2** | **Comparator 3** |
| --- | --- | --- | --- | --- | --- | --- | --- | --- | --- |
| Antunes P. et al, 2019 [1] | Portugal | 90 | Breast cancer | Chemotherapy treatment phase | 2 | Aerobic exercise and resistance training | Usual Care | N/A | N/A |
| Arthuso F.Z. et al, 2021 [2] | Canada | 66 | Breast cancer | Adjuvant therapy phase | 2 | High intensity interval training | Usual Care | N/A | N/A |
| Bourne J.E. et al,2023 [3] | UK | 40 | Prostate and breast cancer | Two timepoints: 1: At diagnosis, 2: Completion of primary cancer treatment | 2 | E‑bike training | Waitlist | N/A | N/A |
| Brunet J. et al, 2020 [4] | Canada | 84 | Breast cancer | Two timepoints: 1 neoadjuvant chemotherapy phase, 2: adjuvant chemotherapy treatment phase | 2 | Aerobic exercise training | Usual Care | N/A | N/A |
| Carraça E.V. et al, 2023 [5] | Portugal | 122 | Breast cancer | Post primary cancer treatment (≥1 month ) | 3 | Aerobic exercise and resistance training | Physical activity counselling | Usual care | N/A |
| Cordier D. et al, 2019 [6] | Switzerland | 45 | Glioma | Post adjuvant therapy | 3 | Aerobic exercise training | Group resistance training | Active control | N/A |
| Cuesta-Vargas A. I. et al, 2016 [7] | Spain | 50 | Testicular cancer | Chemotherapy treatment phase | 2 | Aerobic exercise and resistance training | Control | N/A | N/A |
| De Boniface J. et al, 2022 [8] | Sweden | 712 | Breast cancer | Chemotherapy treatment phase | 2 | High intensity interval training and resistance training | Usual Care | N/A | N/A |
| Díaz‑Balboa E. et al,2021 [9] | Spain | 340 | Breast cancer | Chemotherapy treatment phase | 2 | Aerobic exercise and resistance training | Usual Care | N/A | N/A |
| Dolin T.G. et al, 2021 [10] | Denmark | 100 | Colorectal cancer | Three timepoints: 1: Preoperative, 2: perioperative, 3: postoperative | 2 | Resistance training | Usual Care | N/A | N/A |
| Dubu J. et al, 2022 [11] | France | 186 | Lymphoma | Chemotherapy treatment phase | 2 | Aerobic exercise and resistance training | Usual Care | N/A | N/A |
| Foulkes S.J. et al, 2020 [12] | Australia | 100 | Breast cancer | Three timepoints: 1: Chemotherapy treatment phase, 2: post chemotheraphy treatment, 3: maintenance phase | 2 | multi-component periodised exercise training | Usual Care | N/A | N/A |
| Fournié C. et al, 2020 [13] | France | 70 | Haematological malignancies | Completion of primary cancer treatment (<6 months) | 2 | Heart rate variability biofeedback with aerobic exercise and resistance training | Aerobic exercise and resistance training | N/A | N/A |
| Gal R. et al, 2017 [14] | Netherlands | 166 | Breast cancer | Post primary cancer treatment | 2 | Aerobic exercise and resistance training | Usual Care | N/A | N/A |
| Gentry A.L. et al, 2018 [15] | United States | 254 | Breast cancer | Adjuvant therapy phase | 2 | Aerobic exercise training | Usual Care | N/A | N/A |
| Gokal K. et al, 2015 [16] | UK | 62 | Breast cancer | Neoadjuvant or adjuvant chemotherapy treatment phase | 2 | Aerobic exercise training | Usual Care | N/A | N/A |
| González‐Santos A. et al, 2021 [17] | Spain | 56 | Breast cancer | Two timepoints: Pre adjuvant chemotherapy **OR** 2: Adjuvant chemotherapy phase | 2 | Pre adjuvant aerobic and strength exercises and myofascial and breathing exercises | Adjuvant chemotherapy phase aerobic and strength exercises and myofascial and breathing exercises | N/A | N/A |
| Gonzalo-Encabo P. et al,2022 [18] | United States | 160 | Breast cancer | Post op or post adjuvant treatment (>6 weeks) | 2 | Aerobic exercise and resistance training | Attention Control | N/A | N/A |
| Gothe N.P. et al.,2020 [19] | United States | 75 | Multiple cancer types | Post primary cancer treatment (>1 year) | 3 | Walking intervention | Yoga | Active control | N/A |
| Gouez M. et al, 2022 [20] | France | 30 | Lung cancer | Immune-chemotherapy treatment phase | 2 | Acute aerobic exercise and home-based walking programme | Control Group | N/A | N/A |
| Grigoletto I. et al, 2023 [21] | Brazil | 40 | Lung or head and neck cancer | Chemotherapy treatment phase | 2 | Aerobic exercise and resistance training | Usual Care | N/A | N/A |
| Grimmett et al, 2022 [22] | UK | 1560 | intra-cavity cancer | Preoperative | 4 | High intensity interval training | High intensity interval training and psychological support | Psychological support | Usual care |
| Hart N.H. et al, 2018 [23] | Australia | 40 | Breast cancer with stable osteolytic spinal metastases | Post palliative radiation therapy | 2 | Spinal isometric training with multi-modal training | Usual Care | N/A | N/A |
| Hart N.H. et al, 2017 [24] | Australia | 40 | Prostate cancer with stable sclerotic spinal metastases | Post chemoradiation phase | 2 | Spinal isometric training with multi-modal training | Usual Care | N/A | N/A |
| Hathiramani S. et al, 2019 [25] | UK | 46 | Lymphoma | Post chemotherapy phase (<6 weeks) | 2 | Aerobic exercise and resistance training | Relaxation intervention | N/A | N/A |
| Henkin J.S. et al, 2023 [26] | Brazil | 84 | Breast cancer | Neoadjuvant or adjuvant chemotherapy treatment phase | 3 | Multiple sets resistance training plus aerobic training | Single set resistance training plus aerobic training | Control group | N/A |
| Hernon J. et al, 2021 [27] | UK | 1146 | Colorectal | Preoperative and postoperative | 3 | Hospital Based Supervised Exercise | Home based supported exercise | Usual care | N/A |
| Hyatt A. et al, 2020 [28] | Australia | 30 | Melanoma | Immunotherapy treatment phase | 2 | Aerobic exercise and resistance training | Usual care | N/A | N/A |
| Inbaraj G. et al, 2022 [29] | India | 150 | Breast cancer | Chemotherapy treatment phase | 2 | Yoga | Usual care | N/A | N/A |
| Joly F. et al, 2020 [30] | France | phase II: n = 120; phase III: n = 312 | Multiple cancer types | Post chemotherapy, oral targeted therapy phase | 2 | supervised home-based standardized physical activity program | Control group | N/A | N/A |
| Kang D.W. et al, 2019 [31] | Canada | 66 | Prostate cancer | Active surveillance | 2 | High intensity interval training | Usual care | N/A | N/A |
| Keats M.R. et al, 2022 [32] | Canada | 38 | Glioblastoma | Active oncologic treatment phase | 2 | Resistance training | Usual care | N/A | N/A |
| Kiesl D. et al, 2022 [33] | Austria | 126 | Breast cancer | Neoadjuvant or adjuvant chemotherapy treatment phase | 2 | High intensity interval training | Usual care | N/A | N/A |
| Kotte M. et al, 2023 [34] | Sweden | 200 | Breast, prostate, or colorectal cancer | Post primary treatment | 2 | Aerobic exercise and resistance training | Usual care | N/A | N/A |
| Lavín‑Pérez A.M. et al, 2023 [35] | Spain | 90 | Breast cancer | Post primary treatment (<5 months) | 3 | High intensity exercise | Moderate to high intensity aerobic exercise | Usual care | N/A |
| Lee K. et al, 2022 [36] | United States | 60 | Multiple myeloma | Post hematopoietic stem cell transplant (30–180 days) | 2 | Resistance exercise training | Usual care | N/A | N/A |
| Little R.B. et al, 2024 [37] | United States | 126 | Breast cancer | Post primary treatment >1 year | 2 | Aerobic exercise training | Control group | N/A | N/A |
| Loh K.P. et al, 2022 [38] | United States | 100 | Myeloid neoplasms | Chemotherapy treatment phase | 2 | daily home-based progressive aerobic walking and resistance exercises | Control group | N/A | N/A |
| Loughney L. et al, 2016 [39] | UK | 46 | Rectal cancer | Pre operative and post neoadjuvant treatment | 2 | Aerobic interval training | Usual care | N/A | N/A |
| Luo H. et al, 2021 [40] | Australia | 40 | Pancreatic cancer | Neoadjuvant treatment phase | 2 | Aerobic exercise and resistance training | Usual care | N/A | N/A |
| Martinez Aguirre-Betolaza A. et al, 2024 [41] | Spain | 120 | Breast cancer | Post primary treatment | 3 | Resistance training | Resistance training plus creatine supplementation | Usual care | N/A |
| Martínez-Velilla N. et al, 2023 [42] | Spain | 240 | Multiple cancer types | Inpatient secondary to acute illness | 2 | Aerobic exercise and resistance training | Usual care | N/A | N/A |
| McCourt O. et al, 2020 [43] | UK | 60 | Myeloma | Three timepoints: 1: Pre-transplant, 2: peri-transplant, 3: post-transplant | 2 | Aerobic exercise and resistance training | Usual care | N/A | N/A |
| McIsaac D.I. et al, 2018 [44] | Canada | 200 | Multiple cancer types | Preoperative | 2 | Aerobic exercise, flexibility and resistance training | Control group | N/A | N/A |
| Morielli A.R. et al, 2018 [45] | Canada | 60 | Rectal cancer | Neoadjuvant treatment phase | 2 | High-intensity interval training and moderate intensity training | Usual care | N/A | N/A |
| Murphy K. et al, 2024 [46] | Ireland | 24 | Prostate cancer | Three timepoints: 1: Initiation of ADT treatment OR one-month post-RT OR approximately six months into ADT treatment | 2 | Aerobic exercise training | Resistance exercise training | N/A | N/A |
| Neuzillet C. et al, 2015 [47] | France | 200 | Pancreatic cancer | Chemotherapy treatment phase | 2 | Aerobic exercise and resistance training | Usual care | N/A | N/A |
| Newton R.U. et al, 2018 [48] | Australia | 866 | Prostate cancer | Active hormone therapy phase | 2 | High-intensity aerobic and resistance training | Control group | N/A | N/A |
| Nicol J.L. et al, 2022 [49] | Australia | 60 | Multiple myeloma | Three timepoints: 1 active treatment,2 transplant eligible; active treatment, 3 transplant noneligible; non-active; relapsed | 2 | Aerobic exercise and resistance training | Waitlist control | N/A | N/A |
| Nilsen T.S. et al, 2023 [50] | Norway | 209 | Breast cancer | Survivorship (8-12 years) | 2 | Aerobic exercise training | Usual care | N/A | N/A |
| Oberste M. et al, 2018 [51] | Germany | 118 | Breast cancer | Chemotherapy treatment phase | 2 | High-intensity interval training | Control group | N/A | N/A |
| Patel D.I. et al, 2020 [52] | United States | 30 | Prostate cancer | Active surveillance | 2 | Aerobic exercise and resistance training | Control group | N/A | N/A |
| Patel D.I. et al, 2022 [53] | United States | 30 | Breast cancer | Post chemotherapy treatment (<6 months) | 2 | Resistance training and creatine | Control group | N/A | N/A |
| Poort H. et al, 2017 [54] | Netherlands | 219 | Multiple cancer types | Palliative care | 3 | Aerobic exercise and resistance training | Cognitive and behavioural therapy | Usual care | N/A |
| Porserud A. et al, 2020 [55] | Sweden | 120 | Bladder cancer | Postoperative | 2 | Aerobic exercise and resistance training | Control group | N/A | N/A |
| Postigo-Martin P. et al, 2021 [56] | Spain | 120 | Breast cancer | Pre chemotherapy treatment phase | 2 | Pre chemotherapy phase: Aerobic exercise and resistance training | Chemotherapy phase | N/A | N/A |
| Potiaumpai M. et al, 2023 [57] | United States | 84 | Haematological malignancies | Pre transplant phase | 2 | Aerobic exercise and resistance training | Usual care | N/A | N/A |
| Ramirez-Parada et al, 2022 [58] | Chile | 106 | Breast cancer | Adjuvant chemotherapy phase | 2 | Resistance training | Control group | N/A | N/A |
| Reimer N. et al, 2022 [59] | Germany | 60 | Prostate cancer | Androgen deprivation therapy phase | 2 | Aerobic exercise, impact and resistance training | Control group | N/A | N/A |
| Riani Costa L.A. et al, 2021 [60] | Brazil | 36 | Breast cancer | Post treatment phase | 2 | Canoeing | Control group | N/A | N/A |
| Rodriguez‑Arietaleanizbeaskoa M. et al, 2023 [61] | Spain | 144 | Head and neck cancer | Two timepoints: 1, pre-radiotherapy OR 2:Post-radiotherapy | 3 | Pre-radiotherapy aerobic exercise and resistance training | Post-radiotherapy aerobic exercise and resistance training | Autonomous exercise | N/A |
| Ryu J. et al, 2023 [62] | South Korea | 96 | Breast cancer | Three timepoints: 1 Post-operative, 2: Adjuvant therapy phase,3: Post adjuvant treatment | 2 | 1: Post-operative ROM and resistance training, 2: Adjuvant therapy phase resistance training ,3: Post adjuvant treatment aerobic exercise training | Usual care | N/A | N/A |
| Sheill G. et al, 2020 [63] | Ireland | 78 | Lung and oesophageal cancer | Preoperative | 2 | High intensity interval training | Usual care | N/A | N/A |
| Sheill G. et al, 2017 [64] | Ireland | 200 | Prostate cancer | Stable metastatic disease | 2 | Aerobic exercise training | Usual care | N/A | N/A |
| Sitjar P.H.S. et al, 2024 [65] | Singapore | 50 | Breast cancer | Post primary treatment | 2 | Aerobic exercise and resistance training | Usual care | N/A | N/A |
| Smith L. et al, 2021 [66] | UK | 30 | Bladder cancer | Post diagnosis and into survivorship | 2 | Aerobic exercise, balance and resistance training | Usual care | N/A | N/A |
| Soriando-Maldonado A. et al, 2019 [67] | Spain | 60 | Breast cancer | Post primary treatment (<5 years) | 2 | Aerobic exercise and resistance training | Control group | N/A | N/A |
| Steffens D. et al, 2022 [68] | Australia | 172 | Multiple cancer types | Preoperative | 2 | Aerobic exercise, respiratory muscle training and resistance training | Usual care | N/A | N/A |
| Steffens D. et al, 2018 [69] | Australia | 20 | Gastrointestinal cancers | Preoperative | 2 | Aerobic exercise, respiratory muscle training and resistance training | Usual care | N/A | N/A |
| Toohey K. et al, 2022 [70] | Australia | 160 | Multiple cancer types | Active treatment phase | 2 | Aerobic exercise and resistance training | Usual care | N/A | N/A |
| Touillaud M. et al, 2021 [71] | France | 432 | Breast cancer | Adjuvant treatment phase | 4 | Connected device exercise programme | Therapeutic patient education | Combination of both interventions | Control group |
| Tully R. et al, 2020 [72] | Ireland | 62 | Oesophagogastric cancers | Two timepoints: Preoperative and postoperative | 2 | Aerobic interval training and resistance training | Usual care | N/A | N/A |
| Twomey R. et al, 2018 [73] | Canada | 52 | Multiple cancer types | Post primary treatment ( ≥ 3 months ≤ 5 years) | 2 | Tailored exercise group | Control group | N/A | N/A |
| Van Aperen K. et al, 2023 [74] | Belgium | 150 | Head and neck cancer | Active chemoradiation treatment phase | 2 | Aerobic exercise and resistance training | Usual care | N/A | N/A |
| Van Blarigan E.L. et al, 2023 [75] | USA | 200 | Prostate Cancer | Preoperative and postoperative | 4 | Unsupervised aerobic and resistance training | Dietary intervention only | Exercise and dietary intervention | Usual care |
| Van Vulpen J.K. et al, 2017 [76] | Netherlands | 150 | Oesophageal cancer | Postoperative | 2 | Aerobic exercise and resistance training | Usual care | N/A | N/A |
| Viamonte S.G. et al, 2023 [77] | Portugal | 80 | Multiple cancer types | Post primary treatment | 2 | Centre based Aerobic exercise and resistance training | Community based Aerobic exercise and resistance training | N/A | N/A |
| Vikmoen O. et al, 2022 [78] | Norway | 80 | Breast cancer | Two timepoints: Neoadjuvant and adjuvant | 3 | High intensity aerobic exercise and resistance training | Low to moderate intensity aerobic exercise and resistance training | Usual care | N/A |
| Wilson R. et al, 2023 [79] | United States | 50 | Breast cancer | Chemotherapy treatment phase | 2 | High intensity interval training | Control group | N/A | N/A |
| Winters-Stone K.M. et al, 2021 [80] | United States | 360 | Prostate cancer | Active treatment phase and into survivorship | 3 | Resistance training | Tai Ji Quan training | Stretching | N/A |
| Winters-Stone K.M. et al, 2021 [81] | United States | 294 | Prostate, breast and colorectal cancer | Post primary treatment (>6 weeks) | 3 | Resistance training: Supervised group with partner | Separate supervised group exercise classes for survivors or partners, respectively | Unsupervised home exercise program provided to each partner | N/A |
| Witlox L. et al, 2019 [82] | Netherlands | 180 | Breast cancer | Survivorship (2-4 years) | 2 | Aerobic exercise and resistance training | Usual care | N/A | N/A |
| Woodfiel et al, 2018 [83] | New Zealand | 30 | Multiple cancer types | Preoperative | 2 | High intensity interval training | Usual care | N/A | N/A |
| Yoh K. et al, 2018 [84] | Japan | 34 | Pancreatic cancer | Post primary treatment | 2 | Aerobic exercise training | Usual care | N/A | N/A |
| Yoh K. et al, 2018 [85] | Japan | 34 | Pancreatic cancer | Primary treatment phase | 2 | Aerobic exercise training | Usual care | N/A | N/A |
| Zimmer P. et al, 2016 [86] | Germany | 83 | Acute myeloid leukaemia or myelodysplastic syndrome | Active chemoradiation treatment phase | 3 | Aerobic exercise training | Myofascial training release | Usual care | N/A |

**References**

1. Antunes, P., et al., *Impact of exercise training on cardiotoxicity and cardiac health outcomes in women with breast cancer anthracycline chemotherapy: A study protocol for a randomized controlled trial.* Trials, 2019. **20**(1).

2. Arthuso, F.Z., et al., *Bladder cancer and exeRcise trAining during intraVesical thErapy - The BRAVE trial: A study protocol for a prospective, single-centre, phase II randomised controlled trial.* BMJ open, 2021. **11**(9).

3. Bourne, J.E., et al., *Study protocol for two pilot randomised controlled trials aimed at increasing physical activity using electrically assisted bicycles to enhance prostate or breast cancer survival.* Pilot and Feasibility Studies, 2023. **9**(1).

4. Brunet, J., et al., *Study protocol of the Aerobic exercise and CogniTIVe functioning in women with breAsT cancEr (ACTIVATE) trial: A two-arm, two-centre randomized controlled trial.* BMC cancer, 2020. **20**(1).

5. Carraça, E.V., et al., *Promoting physical activity through supervised vs motivational behavior change interventions in breast cancer survivors on aromatase inhibitors (PAC-WOMAN): protocol for a 3-arm pragmatic randomized controlled trial.* BMC cancer, 2023. **23**(1).

6. Cordier, D., M. Gerber, and S. Brand, *Effects of two types of exercise training on psychological well-being, sleep, quality of life and physical fitness in patients with high-grade glioma (WHO III and IV): Study protocol for a randomized controlled trial.* Cancer Communications, 2019. **39**(1).

7. Cuesta-Vargas, A.I., et al., *Effectiveness of an individualized program of muscular strength and endurance with aerobic training for improving germ cell cancer-related fatigue in men undergoing chemotherapy: EFICATEST study protocol for a randomized controlled trial.* Trials, 2016. **17**(1).

8. de Boniface, J., et al., *Physical exercise during neoadjuvant chemotherapy for breast cancer as a mean to increase pathological complete response rates: Trial protocol of the randomized Neo-ACT trial.* Plos one, 2022. **17**(10 October).

9. Díaz-Balboa, E., et al., *A randomized trial to evaluate the impact of exercise-based cardiac rehabilitation for the prevention of chemotherapy-induced cardiotoxicity in patients with breast cancer: ONCORE study protocol.* BMC cardiovascular disorders, 2021. **21**(1).

10. Dolin, T.G., et al., *Geriatric assessment and intervention in older vulnerable patients undergoing surgery for colorectal cancer: a protocol for a randomised controlled trial (GEPOC trial).* BMC geriatrics, 2021. **21**(1): p. 88.

11. Dubu, J., et al., *Physical Activity Program for the Survival of Elderly Patients With Lymphoma: Study Protocol for Randomized Phase 3 Trial.* JMIR Research Protocols, 2022. **11**(11).

12. Foulkes, S.J., et al., *Exercise as a diagnostic and therapeutic tool for preventing cardiovascular morbidity in breast cancer patients- The BReast cancer EXercise InTervention (BREXIT) trial protocol.* BMC cancer, 2020. **20**(1).

13. Fournié, C., et al., *Adapted physical activity and cardiac coherence in hematologic patients (APACCHE): Study protocol for a randomized controlled trial.* BMC Sports Science, Medicine and Rehabilitation, 2020. **12**(1).

14. Gal, R., et al., *The effects of exercise on the quality of life of patients with breast cancer (the UMBRELLA Fit study): Study protocol for a randomized controlled trial.* Trials, 2017. **18**(1).

15. Gentry, A.L., et al., *Protocol for Exercise Program in Cancer and Cognition (EPICC): A randomized controlled trial of the effects of aerobic exercise on cognitive function in postmenopausal women with breast cancer receiving aromatase inhibitor therapy.* Contemporary clinical trials, 2018. **67**: p. 109-115.

16. Gokal, K., et al., *Can physical activity help to maintain cognitive functioning and psychosocial well-being among breast cancer patients treated with chemotherapy? A randomised controlled trial: study protocol.* BMC Public Health, 2015. **15**: p. 414.

17. González-Santos, Á., et al., *Neurotoxicity prevention with a multimodal program (ATENTO) prior to cancer treatment versus throughout cancer treatment in women newly diagnosed for breast cancer: Protocol for a randomized clinical trial.* Research in nursing & health, 2021. **44**(4): p. 598-607.

18. Gonzalo-Encabo, P., et al., *Reducing Metabolic Dysregulation in Obese Latina and/or Hispanic Breast Cancer Survivors Using Physical Activity (ROSA) Trial: A Study Protocol.* Frontiers in Oncology, 2022. **12**.

19. Gothe, N.P., et al., *Effects of yoga, aerobic, and stretching and toning exercises on cognition in adult cancer survivors: Protocol of the STAY Fit pilot randomized controlled trial.* Trials, 2020. **21**(1).

20. Gouez, M., et al., *Effect of acute aerobic exercise before immunotherapy and chemotherapy infusion in patients with metastatic non-small-cell lung cancer: protocol for the ERICA feasibility trial.* BMJ open, 2022. **12**(4).

21. Grigoletto, I., et al., *Effects of Semisupervised Exercise Training on Health Outcomes in People With Lung or Head and Neck Cancer: Protocol for a Randomized Controlled Trial.* JMIR Research Protocols, 2023. **12**: p. e43547.

22. Grimmett, C., et al., *The Wessex Fit-4-Cancer Surgery Trial (WesFit): A protocol for a factorial-design, pragmatic randomised-controlled trial investigating the effects of a multi-modal prehabilitation programme in patients undergoing elective major intra-cavity cancer surgery.* F1000Research, 2022. **10**.

23. Hart, N.H., et al., *Mechanical suppression of osteolytic bone metastases in advanced breast cancer patients: A randomised controlled study protocol evaluating safety, feasibility and preliminary efficacy of exercise as a targeted medicine.* Trials, 2018. **19**(1).

24. Hart, N.H., et al., *Can exercise suppress tumour growth in advanced prostate cancer patients with sclerotic bone metastases? A randomised, controlled study protocol examining feasibility, safety and efficacy.* BMJ open, 2017. **7**(5).

25. Hathiramani, S., et al., *Relaxation and exercise in lymphoma survivors (REIL study): A randomised clinical trial protocol.* BMC Sports Science, Medicine and Rehabilitation, 2019. **11**(1).

26. Henkin, J.S., et al., *Exercise volume load in women with breast cancer: Study protocol for the ABRACE randomized clinical trial.* Contemporary Clinical Trials Communications, 2023. **31**.

27. Hernon, J., et al., *SupPoRtive Exercise Programmes for Accelerating REcovery after major ABdominal Cancer surgery trial (PREPARE-ABC): Study protocol for a multicentre randomized controlled trial.* Colorectal Disease, 2021. **23**(10): p. 2750-2760.

28. Hyatt, A., et al., *I-Move, a personalised exercise intervention for patients with advanced melanoma receiving immunotherapy: A randomised feasibility trial protocol.* BMJ open, 2020. **10**(2).

29. Inbaraj, G., et al., *Impact of integrated yoga therapy on cognitive impairment and cardiac dysfunction in relation to quality of life in breast cancer patients undergoing chemotherapy: Study protocol for a two-arm randomized controlled trial.* Frontiers in Oncology, 2022. **12**.

30. Joly, F., et al., *Feasibility and efficacy of a supervised home-based physical exercise program for metastatic cancer patients receiving oral targeted therapy: Study protocol for the phase II/III - UNICANCER SdS 01 QUALIOR trial.* BMC cancer, 2020. **20**(1).

31. Kang, D.W., et al., *Exercise duRing active surveillance for prostate cancer-the ERASE trial:A study protocol of a phase II randomised controlled trial.* BMJ open, 2019. **9**(7).

32. Keats, M.R., et al., *The Impact of Resistance Exercise on Muscle Mass in Glioblastoma in Survivors (RESIST): Protocol for a Randomized Controlled Trial.* JMIR Research Protocols, 2022. **11**(5): p. e37709.

33. Kiesl, D., et al., *Protocol for the Exercise, Cancer and Cognition – The ECCO-Study: A Randomized Controlled Trial of Simultaneous Exercise During Neo-/Adjuvant Chemotherapy in Breast Cancer Patients and Its Effects on Neurocognition.* Frontiers in Neurology, 2022. **13**.

34. Kotte, M., et al., *Distance-based delivery of exercise for people treated for breast, prostate or colorectal cancer: a study protocol for a randomised controlled trial of EX-MED Cancer Sweden.* Trials, 2023. **24**(1).

35. Lavín-Pérez, A.M., et al., *High-intensity exercise prescription guided by heart rate variability in breast cancer patients: a study protocol for a randomized controlled trial.* BMC Sports Science, Medicine and Rehabilitation, 2023. **15**(1).

36. Lee, K., et al., *Telehealth exercise to Improve Physical function and frailty in patients with multiple myeloma treated with autologous hematopoietic Stem cell transplantation (TIPS): protocol of a randomized controlled trial.* Trials, 2022. **23**(1).

37. Little, R.B., et al., *Role of Gut Microbe Composition in Psychosocial Symptom Response to Exercise Training in Breast Cancer Survivors (ROME) study: protocol for a randomised controlled trial.* BMJ open, 2024. **14**(5).

38. Loh, K.P., et al., *Protocol for a pilot randomized controlled trial of a mobile health exercise intervention for older patients with myeloid neoplasms (GO-EXCAP 2).* Journal of Geriatric Oncology, 2022. **13**(4): p. 545-553.

39. Loughney, L., et al., *The effects of neoadjuvant chemoradiotherapy and an in-hospital exercise training programme on physical fitness and quality of life in locally advanced rectal cancer patients (The EMPOWER Trial): Study protocol for a randomised controlled trial.* Trials, 2016. **17**(1).

40. Luo, H., et al., *Feasibility and efficacy of a multicomponent exercise medicine programme in patients with pancreatic cancer undergoing neoadjuvant therapy (the EXPAN trial): Study protocol of a dual-centre, two-armed phase i randomised controlled trial.* BMJ Open Gastroenterology, 2021. **8**(1).

41. Martinez Aguirre-Betolaza, A., J. Cacicedo, and A. Castañeda-Babarro, *Creatine Supplementation and Resistance Training in Patients with Breast Cancer (CaRTiC Study): Protocol for a Randomized Controlled Trial.* American Journal of Clinical Oncology: Cancer Clinical Trials, 2024. **47**(4): p. 161-168.

42. Martínez-Velilla, N., et al., *Tailored Prevention of Functional Decline through a Multicomponent Exercise Program in Hospitalized Oncogeriatric Patients: Study Protocol for a Randomized Clinical Trial.* Journal of Nutrition, Health and Aging, 2023. **27**(10): p. 911-918.

43. McCourt, O., et al., *PERCEPT myeloma: A protocol for a pilot randomised controlled trial of exercise prehabilitation before and during autologous stem cell transplantation in patients with multiple myeloma.* BMJ open, 2020. **10**(1).

44. McIsaac, D.I., et al., *PREHAB study: A protocol for a prospective randomised clinical trial of exercise therapy for people living with frailty having cancer surgery.* BMJ open, 2018. **8**(6).

45. Morielli, A.R., et al., *Exercise during and after neoadjuvant rectal cancer treatment (the EXERT trial): Study protocol for a randomized controlled trial.* Trials, 2018. **19**(1).

46. Murphy, K., et al., *A comparison of aerobic- and resistance-emphasised exercise on cardiometabolic health and quality of life in men receiving androgen deprivation therapy for prostate cancer: Protocol for a feasibility trial.* Contemporary clinical trials, 2024. **136**.

47. Neuzillet, C., et al., *Rationale and design of the Adapted Physical Activity in advanced Pancreatic Cancer patients (APACaP) GERCOR (Groupe Coopérateur Multidisciplinaire en Oncologie) trial: Study protocol for a randomized controlled trial.* Trials, 2015. **16**(1).

48. Newton, R.U., et al., *Intense Exercise for Survival among Men with Metastatic Castrate-Resistant Prostate Cancer (INTERVAL-GAP4): A multicentre, randomised, controlled phase III study protocol.* BMJ open, 2018. **8**(5).

49. Nicol, J.L., et al., *An Individualized Exercise Intervention for People with Multiple Myeloma—Study Protocol of a Randomized Waitlist-Controlled Trial.* Current Oncology, 2022. **29**(2): p. 901-923.

50. Nilsen, T.S., et al., *Effects of Aerobic Exercise on Cardiorespiratory Fitness, Cardiovascular Risk Factors, and Patient-Reported Outcomes in Long-Term Breast Cancer Survivors: Protocol for a Randomized Controlled Trial.* JMIR Research Protocols, 2023. **12**: p. e45244.

51. Oberste, M., et al., *Protocol for the "chemobrain in Motion - Study" (CIM - Study): A randomized placebo-controlled trial of the impact of a high-intensity interval endurance training on cancer related cognitive impairments in women with breast cancer receiving first-line chemotherapy.* BMC cancer, 2018. **18**(1).

52. Patel, D.I., et al., *A randomized controlled trial of a home-based exercise program on prognostic biomarkers in men with prostate cancer: A study protocol.* Contemporary Clinical Trials Communications, 2020. **20**.

53. Patel, D.I., et al., *Exercise and Creatine Supplementation to Augment the Adaptation of Exercise Training Among Breast Cancer Survivors Completing Chemotherapy: Protocol for an Open-label Randomized Controlled Trial (the THRIVE Study).* JMIR Research Protocols, 2022. **11**(4): p. e26827.

54. Poort, H., et al., *Study protocol of the TIRED study: A randomised controlled trial comparing either graded exercise therapy for severe fatigue or cognitive behaviour therapy with usual care in patients with incurable cancer.* BMC cancer, 2017. **17**(1).

55. Porserud, A., et al., *The CanMoRe trial - Evaluating the effects of an exercise intervention after robotic-assisted radical cystectomy for urinary bladder cancer: The study protocol of a randomised controlled trial.* BMC cancer, 2020. **20**(1).

56. Postigo-Martin, P., et al., *Attenuating Treatment-Related Cardiotoxicity in Women Recently Diagnosed With Breast Cancer via a Tailored Therapeutic Exercise Program: Protocol of the ATOPE Trial.* Physical Therapy, 2021. **101**(3).

57. Potiaumpai, M., et al., *IMPROVE-BMT: A protocol for a pilot randomised controlled trial of prehabilitation exercise for adult haematopoietic stem cell transplant recipients.* BMJ open, 2023. **13**(1).

58. Ramírez-Parada, K., et al., *Effect of Supervised Resistance Training on Arm Volume, Quality of Life and Physical Perfomance Among Women at High Risk for Breast Cancer-Related Lymphedema: A Study Protocol for a Randomized Controlled Trial (STRONG-B).* Frontiers in Oncology, 2022. **12**.

59. Reimer, N., et al., *Influence of a 12-month supervised, intensive resistance, aerobic and impact exercise intervention on muscle strength in prostate cancer patients undergoing anti-hormone therapy: Study protocol for the randomized, controlled Burgdorf study.* Contemporary clinical trials, 2022. **114**.

60. Riani Costa, L.A., et al., *The influence of a supervised group exercise intervention combined with active lifestyle recommendations on breast cancer survivors’ health, physical functioning, and quality of life indices: study protocol for a randomized and controlled trial.* Trials, 2021. **22**(1).

61. Rodriguez-Arietaleanizbeaskoa, M., et al., *Protocol for the SEHNeCa randomised clinical trial assesing Supervised Exercise for Head and Neck Cancer patients.* BMC cancer, 2023. **23**(1).

62. Ryu, J., et al., *Effect of a 1-year tailored exercise program according to cancer trajectories in patients with breast cancer: study protocol for a randomized controlled trial.* BMC cancer, 2023. **23**(1).

63. Sheill, G., et al., *Preoperative exercise to improve fitness in patients undergoing complex surgery for cancer of the lung or oesophagus (PRE-HIIT): Protocol for a randomized controlled trial.* BMC cancer, 2020. **20**(1).

64. Sheill, G., et al., *The ExPeCT (Examining Exercise, Prostate Cancer and Circulating Tumour Cells) trial: Study protocol for a randomised controlled trial.* Trials, 2017. **18**(1).

65. Sitjar, P.H.S., et al., *Combined aerobic and strength exercise training on biological ageing in Singaporean breast cancer patients: protocol for the Breast Cancer Exercise Intervention (BREXINT) Pilot Study.* GeroScience, 2024.

66. Smith, L., et al., *The CADENCE pilot trial – Promoting physical activity in bladder cancer survivors: A protocol paper.* Contemporary Clinical Trials Communications, 2021. **22**.

67. Soriano-Maldonado, A., A. Carrera-Ruiz, and D.M. Díez-Fernández, *Effects of a 12-week resistance and aerobic exercise program on muscular strength and quality of life in breast cancer survivors: Study protocol for the EFICAN randomized controlled trial (vol 98, e17625, 2019).* Medicine, 2019. **98**(49).

68. Steffens, D., et al., *PRehabllitatiOn with pReoperatIve exercise and educaTion for patients undergoing major abdominal cancer surgerY: protocol for a multicentre randomised controlled TRIAL (PRIORITY TRIAL).* BMC cancer, 2022. **22**(1).

69. Steffens, D., et al., *Feasibility and acceptability of PrE-operative Physical Activity to improve patient outcomes After major cancer surgery: Study protocol for a pilot randomised controlled trial (PEPA Trial).* Trials, 2018. **19**(1).

70. Toohey, K., et al., *Towards best practice in the delivery of prescribed exercise via telehealth for individuals diagnosed with cancer: A randomised controlled trial protocol.* Contemporary clinical trials, 2022. **119**.

71. Touillaud, M., et al., *Connected device and therapeutic patient education to promote physical activity among women with localised breast cancer (DISCO trial): Protocol for a multicentre 2×2 factorial randomised controlled trial.* BMJ open, 2021. **11**(9).

72. Tully, R., et al., *The effect of a pre- And post-operative exercise programme versus standard care on physical fitness of patients with oesophageal and gastric cancer undergoing neoadjuvant treatment prior to surgery (The PERIOP-OG Trial): Study protocol for a randomised controlled trial.* Trials, 2020. **21**(1).

73. Twomey, R., et al., *Tailored exercise interventions to reduce fatigue in cancer survivors: Study protocol of a randomized controlled trial.* BMC cancer, 2018. **18**(1).

74. Van Aperen, K., et al., *EffEx-HN trial: study protocol for a randomized controlled trial on the EFFectiveness and feasibility of a comprehensive supervised EXercise program during radiotherapy in Head and Neck cancer patients on health-related quality of life.* Trials, 2023. **24**(1).

75. Van Blarigan, E.L., et al., *Protocol for a 4-arm randomized controlled trial testing remotely delivered exercise-only, diet-only, and exercise + diet interventions among men with prostate cancer treated with radical prostatectomy (Prostate 8-II).* Contemporary clinical trials, 2023. **125**.

76. van Vulpen, J.K., et al., *Physical ExeRcise Following Esophageal Cancer Treatment (PERFECT) study: design of a randomized controlled trial.* BMC Cancer, 2017. **17**(1): p. 552.

77. Viamonte, S.G., et al., *Impact of a COmprehensive cardiac REhabilitation framework among high cardiovascular risk cancer survivors: Protocol for the CORE trial.* International Journal of Cardiology, 2023. **371**: p. 384-390.

78. Vikmoen, O., et al., *Effects of High and Low-To-Moderate Intensity Exercise During (Neo-) Adjuvant Chemotherapy on Muscle Cells, Cardiorespiratory Fitness, and Muscle Function in Women With Breast Cancer: Protocol for a Randomized Controlled Trial.* JMIR Research Protocols, 2022. **11**(11): p. e40811.

79. Wilson, R., et al., *Improving Cognitive Function Through High-Intensity Interval Training in Breast Cancer Patients Undergoing Chemotherapy: Protocol for a Randomized Controlled Trial.* JMIR Research Protocols, 2023. **12**: p. e39740.

80. Winters-Stone, K.M., et al., *Protocol for GET FIT Prostate: a randomized, controlled trial of group exercise training for fall prevention and functional improvements during and after treatment for prostate cancer.* Trials, 2021. **22**(1).

81. Winters-Stone, K.M., et al., *Study protocol for the Exercising Together© trial: a randomized, controlled trial of partnered exercise for couples coping with cancer.* Trials, 2021. **22**(1).

82. Witlox, L., et al., *Effect of physical exercise on cognitive function and brain measures after chemotherapy in patients with breast cancer (PAM study): Protocol of a randomised controlled trial.* BMJ open, 2019. **9**(6).

83. Woodfiel, J., et al., *Protocol, and practical challenges, for a randomised controlled trial comparing the impact of high intensity interval training against standard care before major abdominal surgery: study protocol for a randomised controlled trial.* Trials, 2018. **19**.

84. Yoh, K., et al., *Effect of exercise therapy on sarcopenia in pancreatic cancer: A study protocol for a randomised controlled trial.* BMJ Open Gastroenterology, 2018. **5**(1).

85. Yoh, K., et al., *Implication of exercise interventions on sleep disturbance in patients with pancreatic cancer: A study protocol for a randomised controlled trial.* BMJ Open Gastroenterology, 2018. **5**(1).

86. Zimmer, P., et al., *Impact of aerobic exercise training during chemotherapy on cancer related cognitive impairments in patients suffering from acute myeloid leukemia or myelodysplastic syndrome - Study protocol of a randomized placebo-controlled trial.* Contemporary clinical trials, 2016. **49**: p. 1-5.
